# Supplementary material for: Grinding-induced supramolecular charge-transfer assemblies with switchable vapochromism toward haloalkane isomers
Source: Nat Commun. 2023 Sep 23;14:5954. doi: 10.1038/s41467-023-41713-9 (PMC10517982; doi:10.1038/s41467-023-41713-9)
Supplement: Supplementary file 1 — Supplementary Information [file 41467_2023_41713_MOESM1_ESM.pdf]

Supplementary Information

Grinding-Induced Supramolecular Charge-Transfer Assemblies with Switchable Vapochromism Toward Haloalkane Isomers

*Jia-Rui Wu,[1,2] Gengxin Wu,[1] Dongxia Li,[1] Meng-Hao Li,[1] Yan Wang,[1] and Ying-Wei Yang[1]**

[1]International Joint Research Laboratory of Nano-Micro Architecture Chemistry, College of Chemistry, Jilin University, 2699 Qianjin Street, Changchun 130012, P. R. China.

[2]Key Laboratory of Automobile Materials of Ministry of Education, College of Materials Science and Engineering, Jilin University, 5988 Renmin Street, Changchun 130025, P. R. China.

*Email: ywyang@jlu.edu.cn

Contents

1. Supplementary Methods....2
2. Supplementary Discussion....3
    2.1 Investigation of Host−Guest CT Interactions in Solution....3
    2.2. Vapochromic Study with Single-Component Bromoalkane Isomer....7
    2.3. X-Ray Crystallography....22
    2.4. Mechanism Study of the On-off Type Vapochromic Behavior....30
    2.5. Recyclability of EtP5-NBNα and EtP5-DNBα....46
3. Supplementary References....49

1. Supplementary Methods

Starting materials and reagents including NBN, DNB, 1-BPR, 2-BPR, 1-BBU, 2-BBU, 1-BPE and 2-BPE were purchased from commercial suppliers and used without further purification unless stated otherwise. Compound EtP5 was prepared according to a previous reported literature.[1] ^{1}H and ^{13}C NMR spectra were recorded at 298 K on a Bruker AVANCEIII 400-MHz instrument at room temperature. Chemical shifts were referenced to tetramethylsilane. Powder X-ray diffraction (PXRD) measurements were collected on a PANalytical B.V. Empyrean powder diffractometer operating at 40 kV/30 mA using the Cu Kα line (λ = 1.5418 Å), and data were measured over the range 5° to 40° in 5°/min steps over 7 min. Single-crystal X-ray diffraction data were collected by a Bruker D8 Venture diffractometer equipped with a PHOTON 100 CMOS detector, using Ga-Kα radiation (λ = 1.34139 Å) and Mo-Kα radiation (λ = 0.71073 Å). The structures were solved with SHELXT program[2] using Direct Methods or Intrinsic Phasing; refined by full-matrix least-squares on $|F|^2$ by SHELXL;[3] and interfaced through the program OLex2.[4] Thermogravimetric analysis (TGA) and differential scanning calorimetry (DSC) experiment were carried out using a simultaneous thermal analyzer 449 F3 analyzer (NETZSCH Instruments) with an automated vertical overhead thermobalance. The samples were heated at 10 °C/min using N_2 as the protective gas. Fluorescent titration experiments were performed on a Shimadzu RF- 5301PC spectrometer. UV-vis spectra in solution were collected on a Shimadzu UV-2550 spectrometer. Solid-state UV-visible spectra were measured by a reflectance mode on a PerkinElmer Lambda950 spectrometer from 200 to 800 nm with $BaSO_4$ as a reference. Energy-minimized structure and electrostatic potential surface (EPS) were calculated by density functional theory (DFT) using the B3LYP hybrid function combined with 6-31G(d,p) basis set under Gaussian G09. Using single crystal superstructures as input files, independent gradient model (IGM)[5] analyses were carried out by Multiwfn 3.6 program[6] through function 20 (visual study of weak interaction) and visualized using VMD software.[7] Activated host−guest D-A complexes, referred to as EtP5-NBNα and EtP5-DNBα, were prepared by grinding the desolvated powder of EtP5 with NBN and DNB solids in a 1:3 molar ratio, respectively. For each solid-vapor contact experiment, an open 0.5 mL vial containing 3 mg of EtP5-NBNα (or EtP5-DNBα) was placed in a sealed 2 mL vial containing 0.01 mL of single-component bromoalkane isomer or 1:1 mixture of bromoalkane isomers. Relative uptake amounts in EtP5-NBNα and EtP5-DNBα were determined by ^{1}H

NMR integrals of corresponding proton signals by completely dissolving the mixture powders in $CDCl_3$, respectively. Desorption experiments after saturation were carried out by TGA and DSC.

2. Supplementary Discussion

2.1 Investigation of Host−Guest CT Interactions in Solution

2.1.1 NMR Data of the Complexes

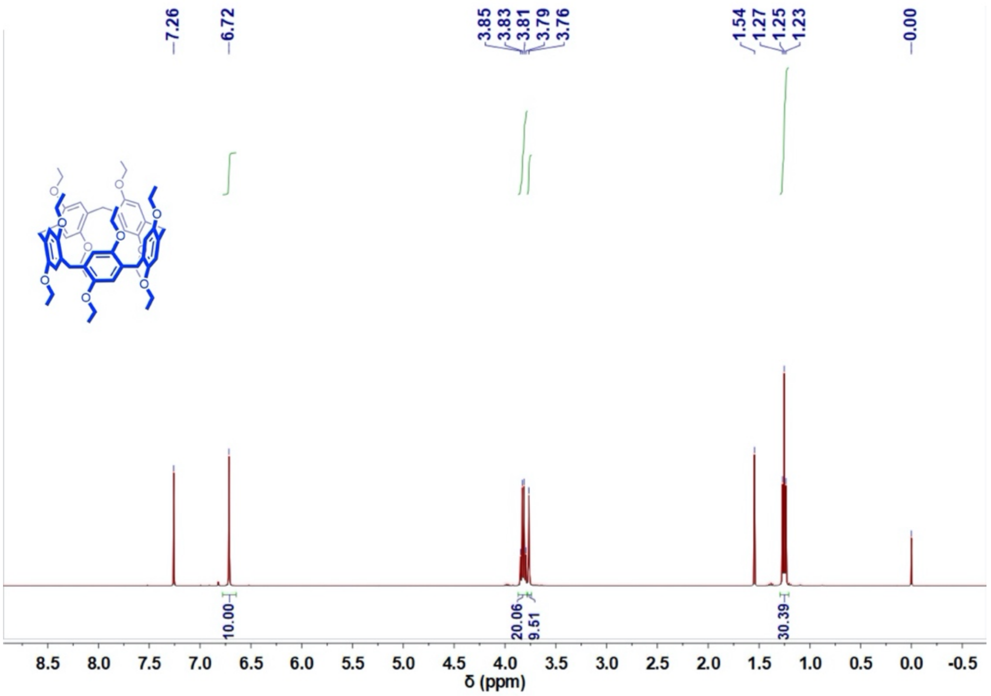

Supplementary Figure 1. ^{1}H NMR spectrum (400 MHz, 298 K, $CDCl_3$) of EtP5.

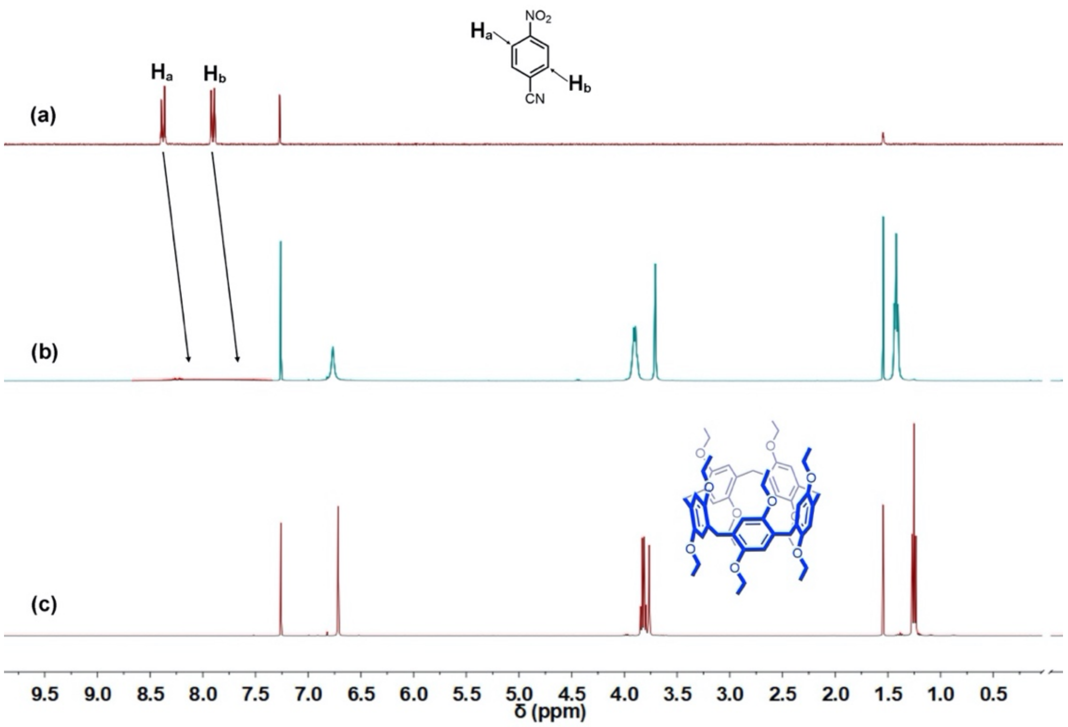

Supplementary Figure 2. ^{1}H NMR spectra (400 MHz, 298 K, $CDCl_3$): (a) free NBN; (b) EtP5 (5 mM) and NBN (15 mM), that is EtP5-NBNα; (c) free EtP5 (5 mM).

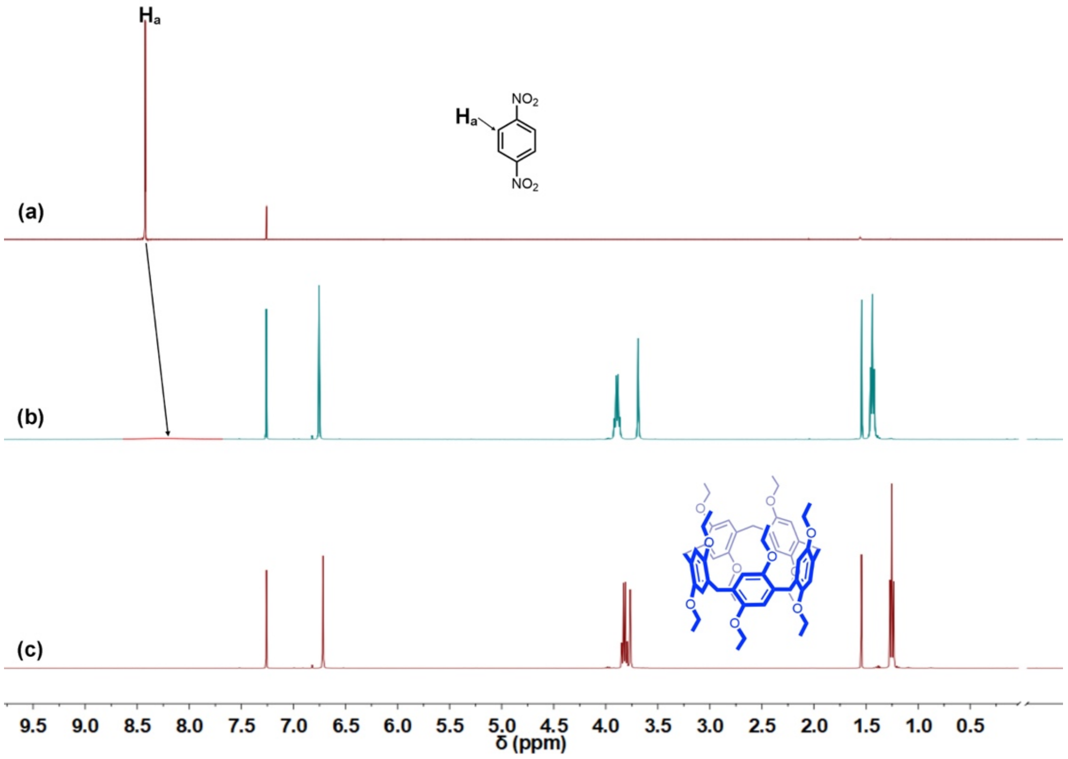

Supplementary Figure 3. ^{1}H NMR spectra (400 MHz, 298 K, $CDCl_3$): (a) free DNB; (b) EtP5 (5 mM) and DNB (15 mM), that is EtP5-DNBα; (c) free EtP5 (5 mM).

2.1.2 Determination of the Association Constants.

Fluorescence and NMR titration curve-fitting and association constant values were calculated by employing the *BindFit* program developed by Prof. Pall Thordarson of UNSW.[8,9] This program employs a nonlinear least-squares regression analysis and is available free of cost online through the following link: http://supramolecular.org/. All of the titration experiments have been repeated three times, and the average values and standard deviations are given in Supplementary Tables 1 and 2. Errors are smaller than ±5%. The figures of one of the three repeated experiments were shown in Fig. 2 and Supplementary Figs. 4-6.

Supplementary Table 1. Binding Constants (K_a)[a] for EtP5 with NBN and DNB at 298 K.

Guests	Solvents	K_a (M^{-1})	Binding Stoichiometry[b]
NBN	$CHCl_3$	11238 ± 459	1:1
DNB	$CHCl_3$	18086 ± 21	1:1

[a] The K_a values were determined by fluorescence titration methods.

[b] 1:1 Binding stoichiometry was chosen in the *BindFit* program.

Supplementary Table 2. Binding Constants (K_a)[a] for EtP5 with 1-BBU[10], 1-BPE, 2-BBU and 2-BPE at 298 K.

Guests	Solvents	K_a (M^{-1})	Binding Stoichiometry[b]
1-BBU	$CHCl_3$	52 ± 4	1:1
2-BBU	$CHCl_3$	[c]	1:1
1-BPE	$CHCl_3$	70 ± 6	1:1
2-BPE	$CHCl_3$	[c]	1:1

[a] The K_a values were determined by NMR titration methods.

[b] 1:1 Binding stoichiometry was chosen in the *BindFit* program.

[c] No interactions were found or at least the association constants were too small (<10 M^{-1}) to be accurately calculated.

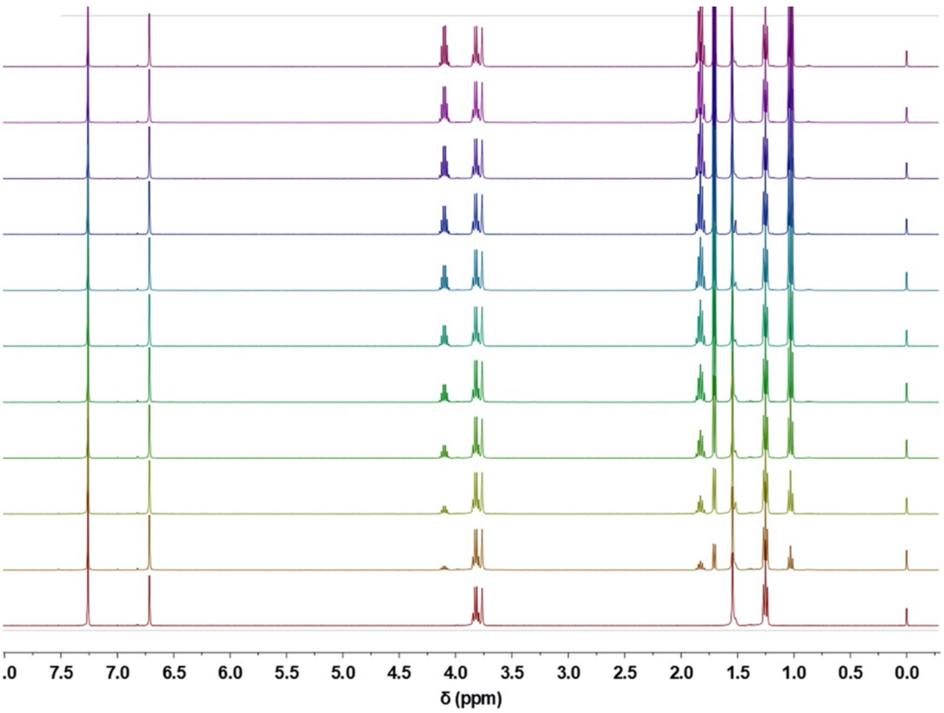

Supplementary Figure 4. ^{1}H NMR spectra (400 MHz, $CDCl_3$, 298 K) of EtP5A at a concentration of 1.0 mM upon addition of 2-BBU. From bottom to top, the concentration of 2-BBU was 0, 2, 4, 6, 8, 10, 12, 14, 16, 18 and 20 mM.

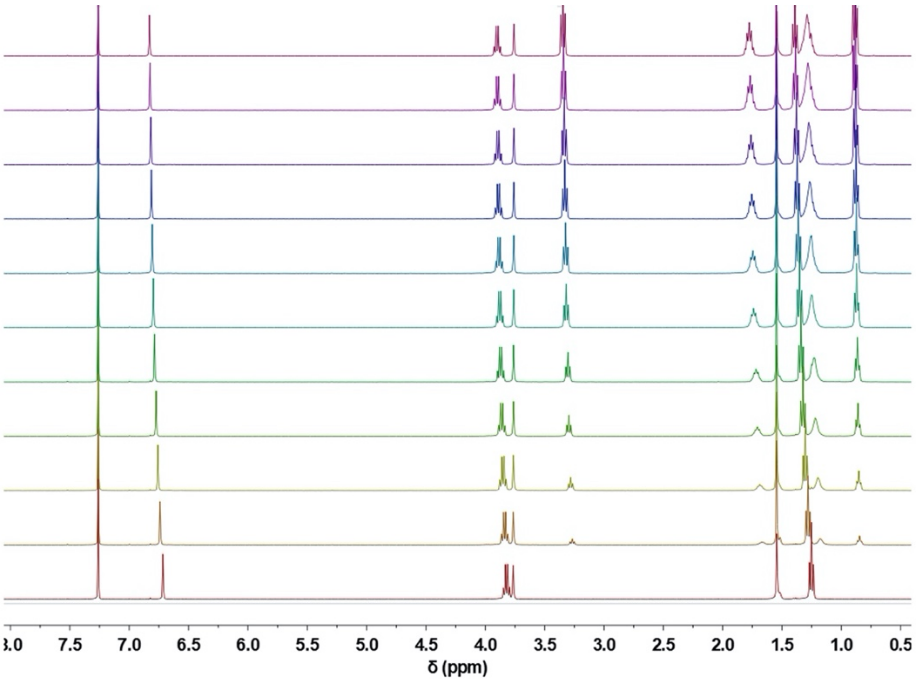

Supplementary Figure 5. ^{1}H NMR spectra (400 MHz, $CDCl_3$, 298 K) of EtP5A at a concentration of 1.0 mM upon addition of 1-BPE. From bottom to top, the concentration of 1-BPE was 0, 2, 4, 6, 8, 10, 12, 14, 16, 18 and 20 mM.

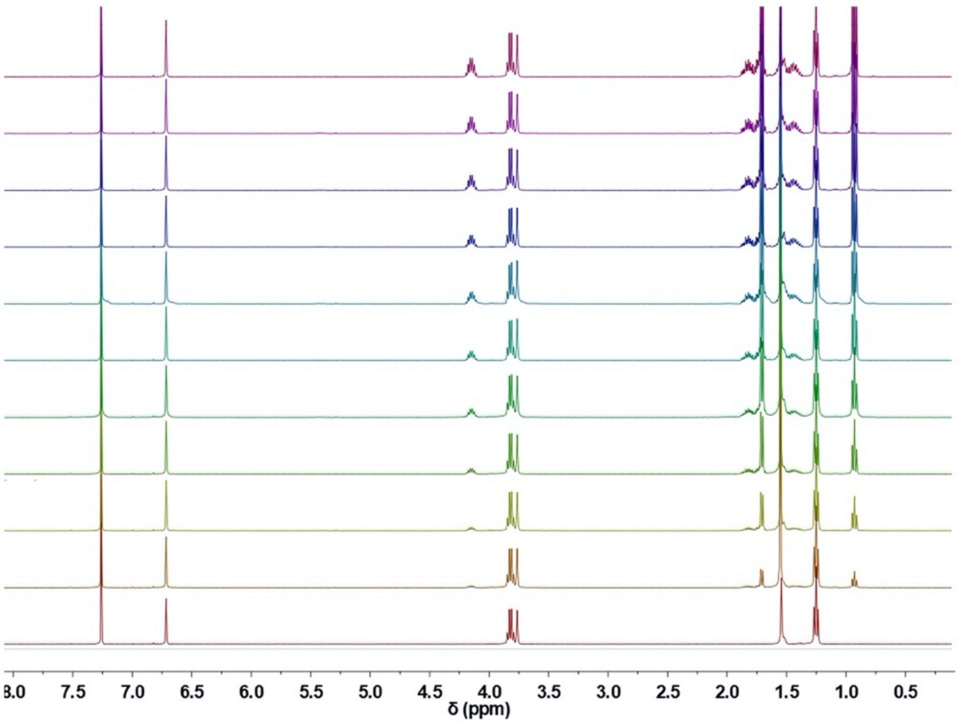

Supplementary Figure 6. ^{1}H NMR spectra (400 MHz, $CDCl_3$, 298 K) of EtP5A at a concentration of 1.0 mM upon addition of 2-BPE. From bottom to top, the concentration of 2-BPE was 0, 2, 4, 6, 8, 10, 12, 14, 16, 18 and 20 mM.

2.2. Vapochromic Study with Single-Component Bromoalkane Isomer

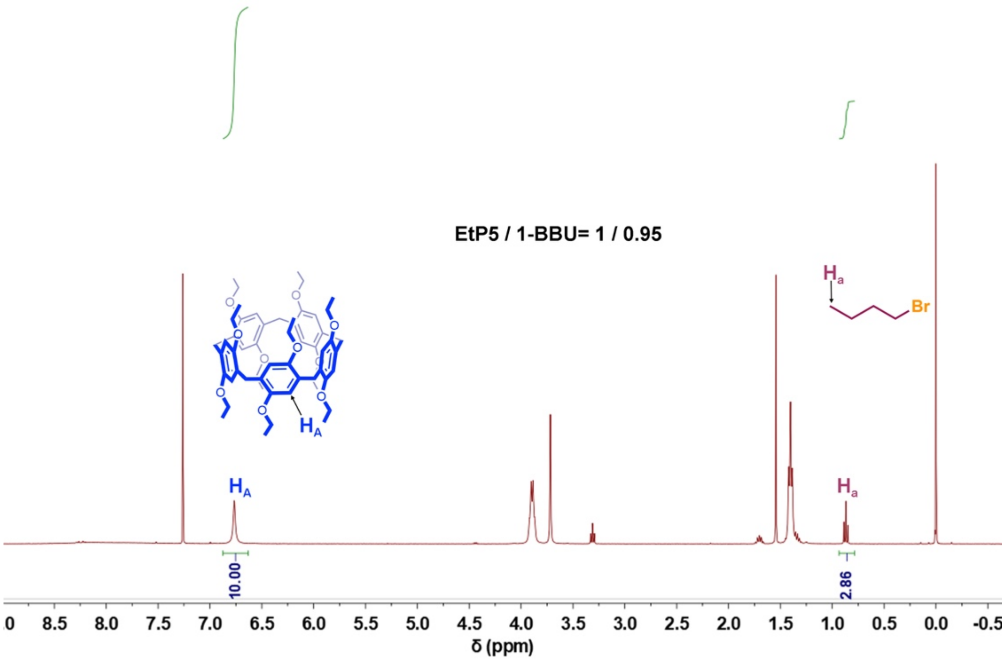

Supplementary Figure 7. ^{1}H NMR spectrum (400 MHz, $CDCl_3$, 298 K) of EtP5-NBNα after exposure to 1-BBU vapor.

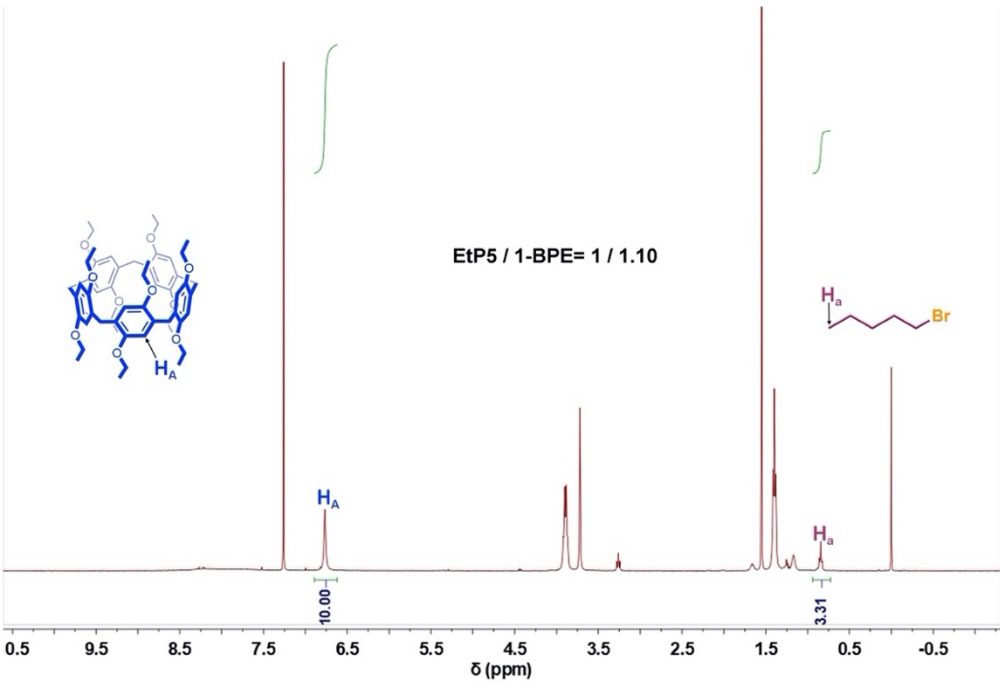

Supplementary Figure 8. ^{1}H NMR spectrum (400 MHz, $CDCl_3$, 298 K) of EtP5-NBNα after exposure to 1-BPE vapor.

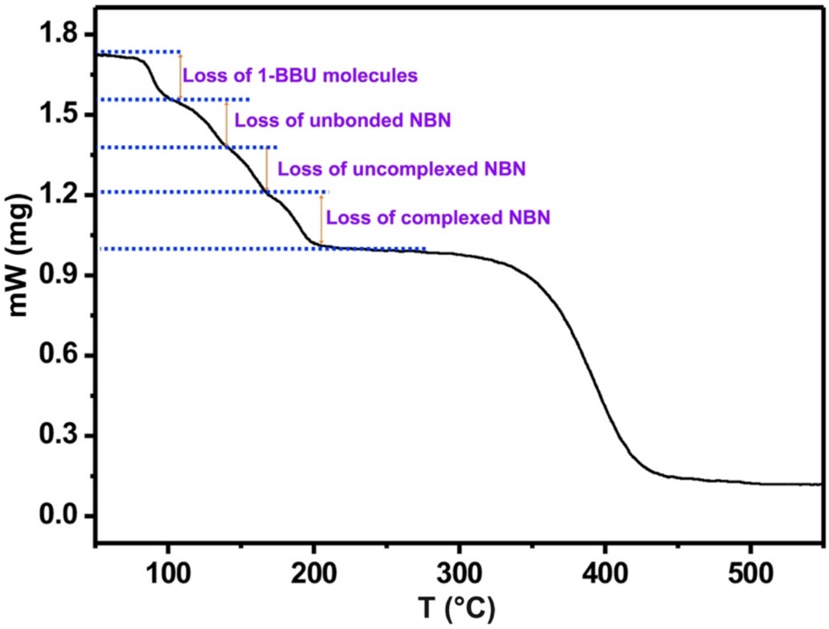

Supplementary Figure 9. TGA of EtP5-NBNα after exposure to 1-BBU vapor.

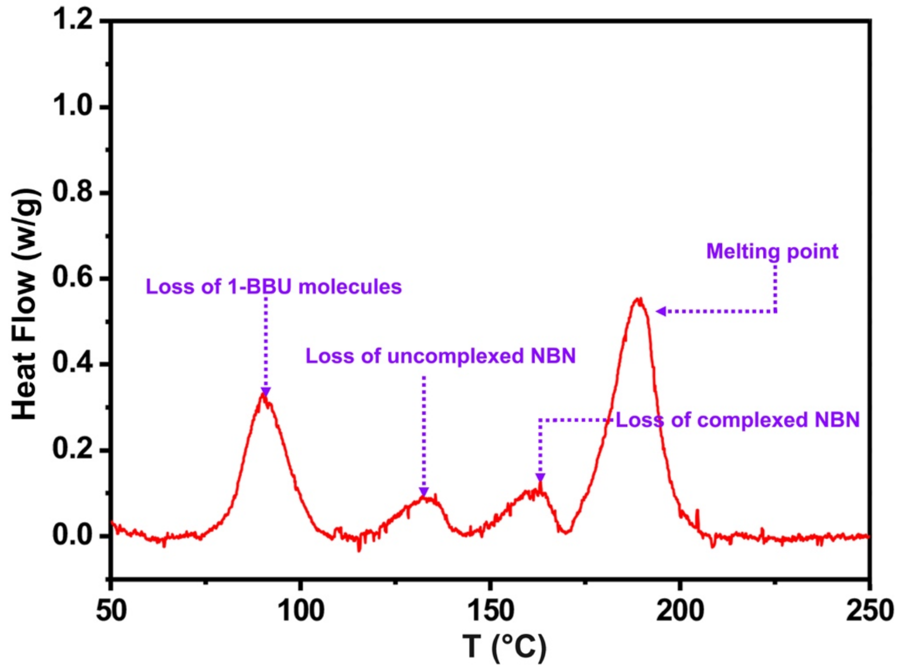

Supplementary Figure 10. DSC trace of EtP5-NBNα after exposure to 1-BBU vapor.

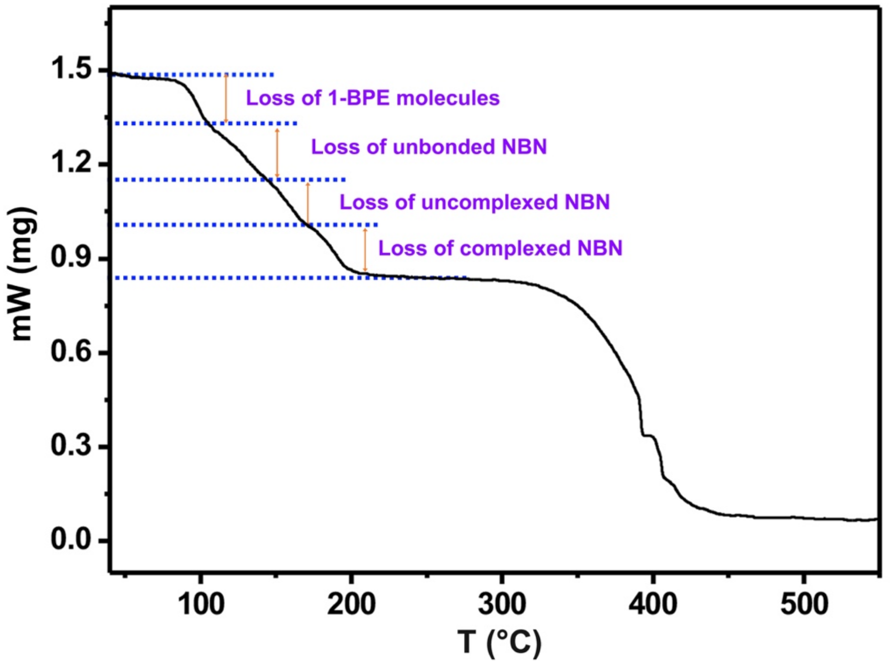

Supplementary Figure 11. TGA of EtP5-NBNα after exposure to 1-BPE vapor.

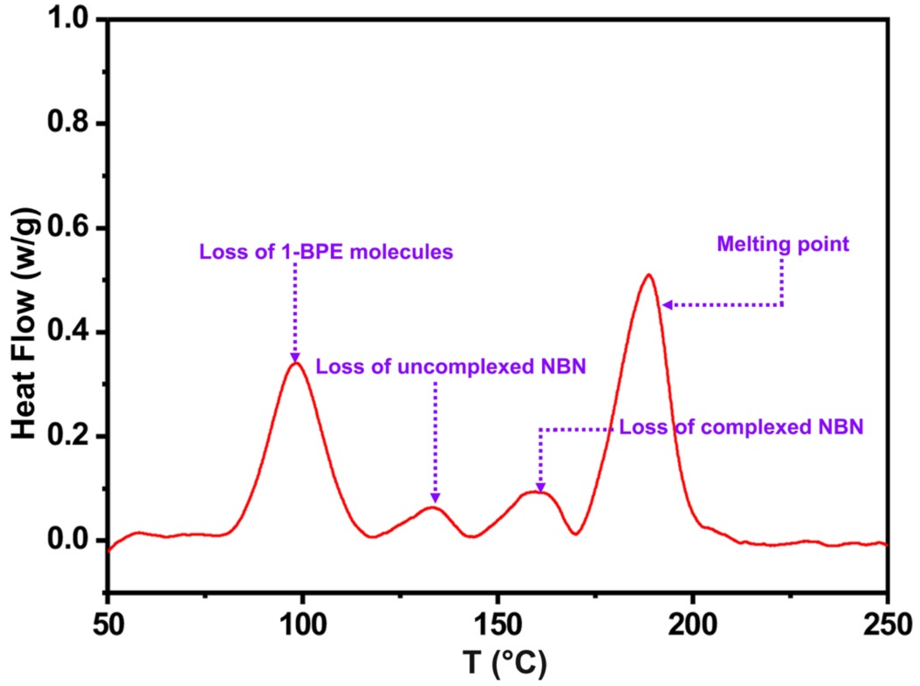

Supplementary Figure 12. DSC trace of EtP5-NBNα after exposure to 1-BPE vapor.

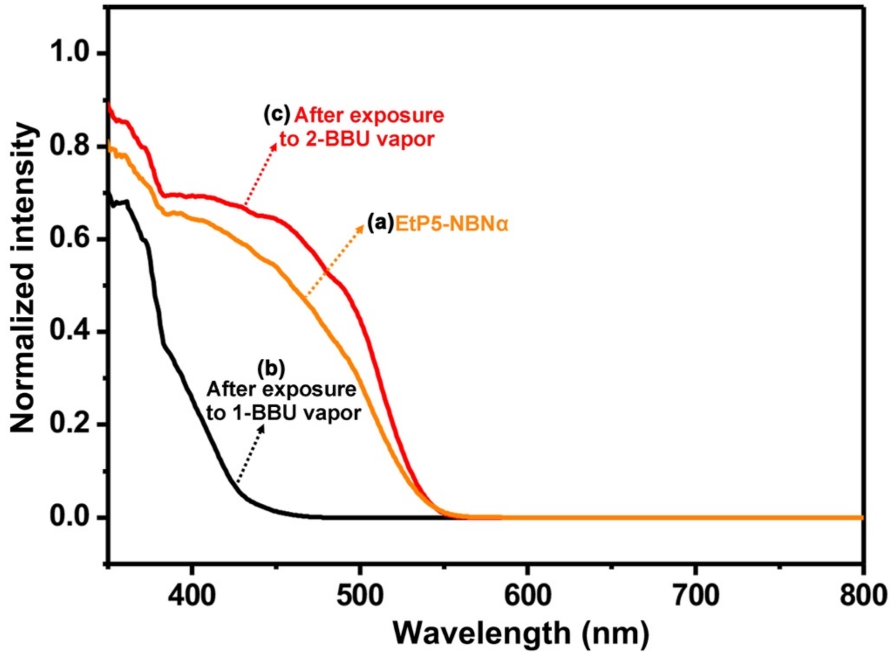

Supplementary Figure 13. Normalized solid-state UV/Vis absorption spectra: (a) original EtP5-NBNα, after exposure to (b) 1-BBU vapor, and (c) 2-BBU vapor.

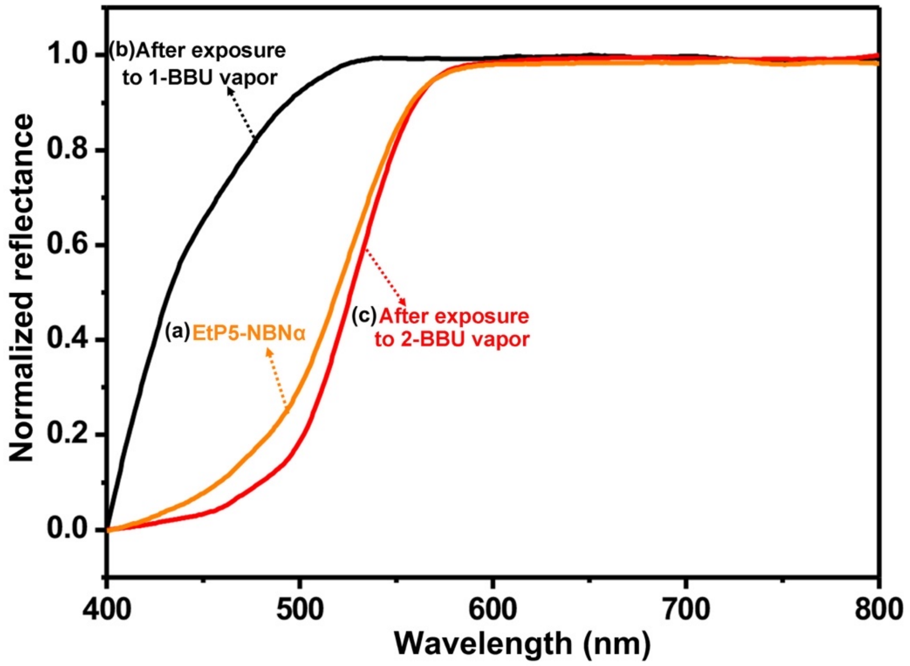

Supplementary Figure 14. Normalized solid-state UV/Vis diffuse reflection spectra: (a) original EtP5-NBNα, after exposure to (b) 1-BBU vapor, and (c) 2-BBU vapor.

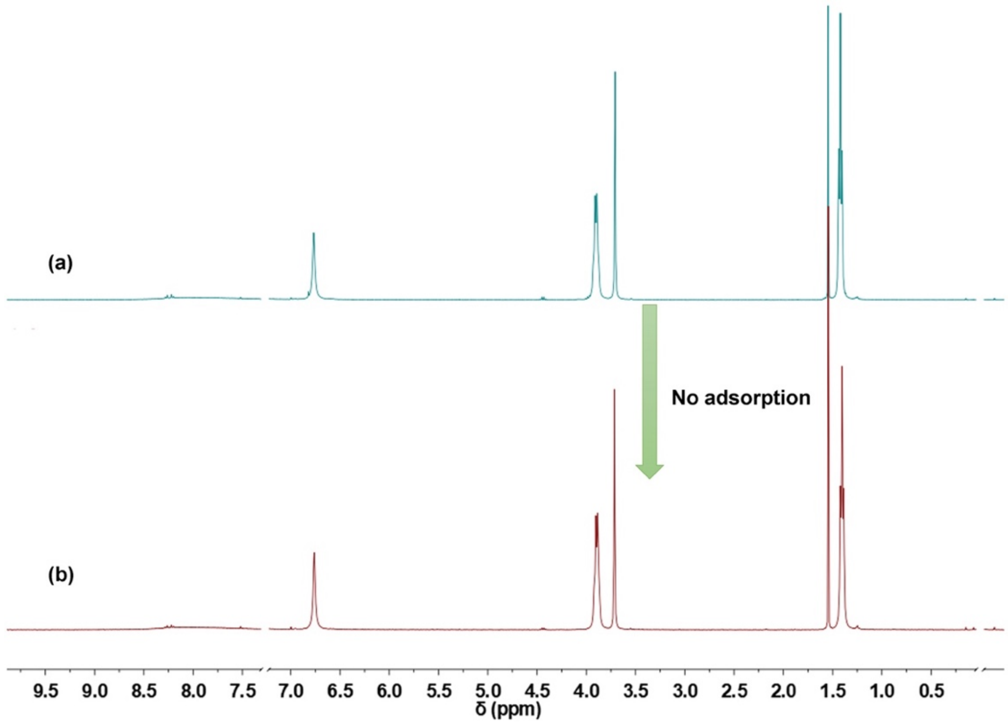

Supplementary Figure 15. ^{1}H NMR spectrum (400 MHz, $CDCl_3$, 298 K) of EtP5-NBNα (a) before and (b) after exposure to 2-BBU vapor.

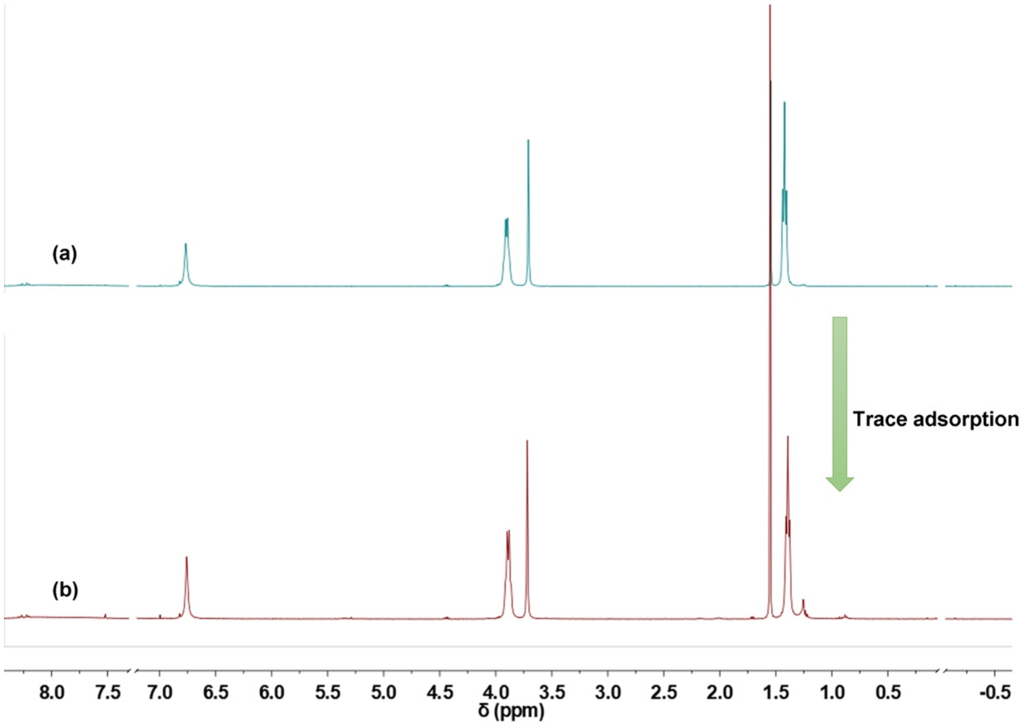

Supplementary Figure 16. ^{1}H NMR spectrum (400 MHz, $CDCl_3$, 298 K) of EtP5-NBNα (a) before and (b) after exposure to 2-BPE vapor.

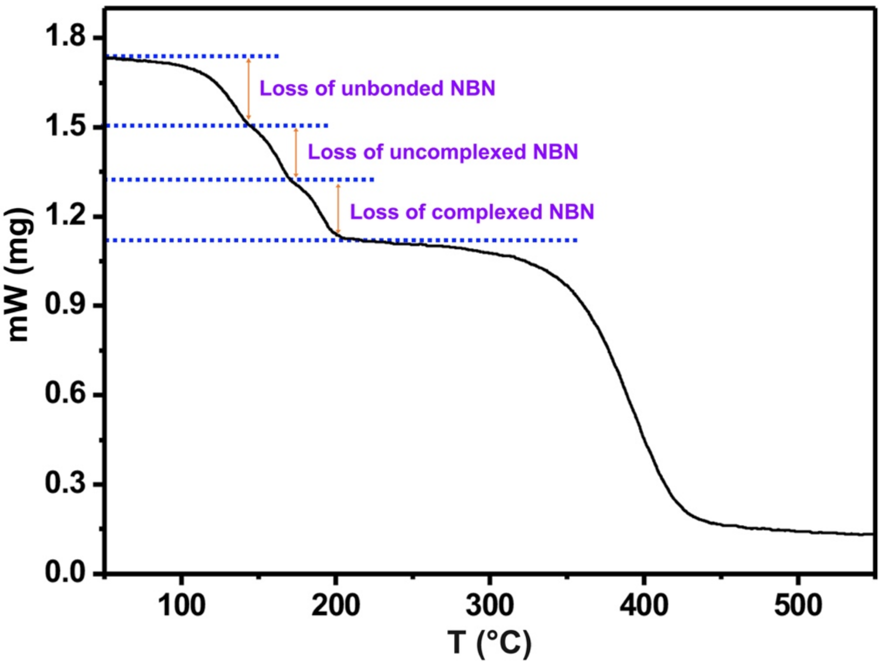

Supplementary Figure 17. TGA of EtP5-NBNα after exposure to 2-BBU vapor.

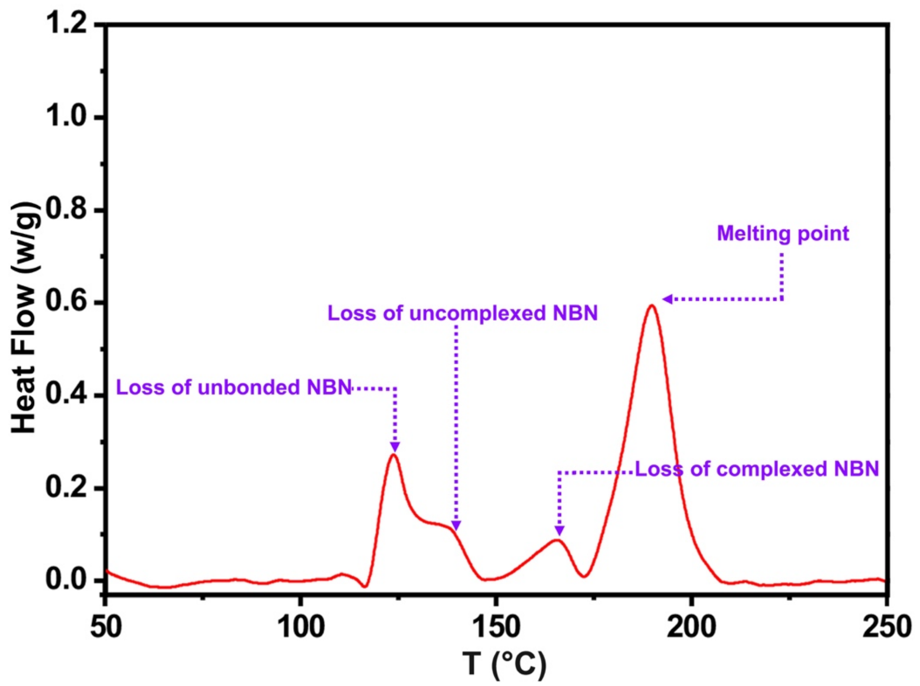

Supplementary Figure 18. DSC trace of EtP5-NBNα after exposure to 2-BBU vapor.

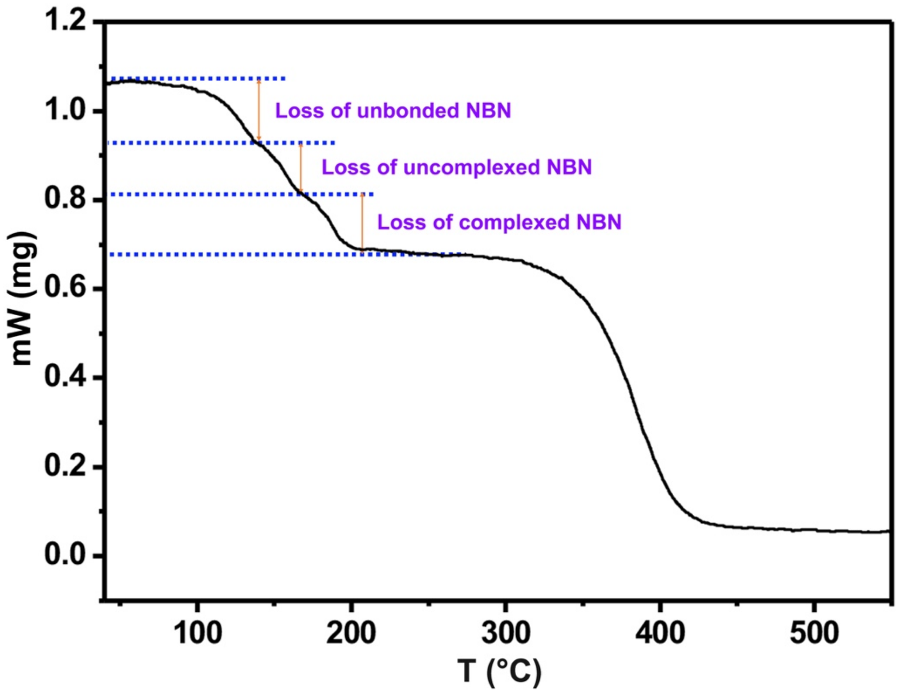

Supplementary Figure 19. TGA of EtP5-NBNα after exposure to 2-BPE vapor.

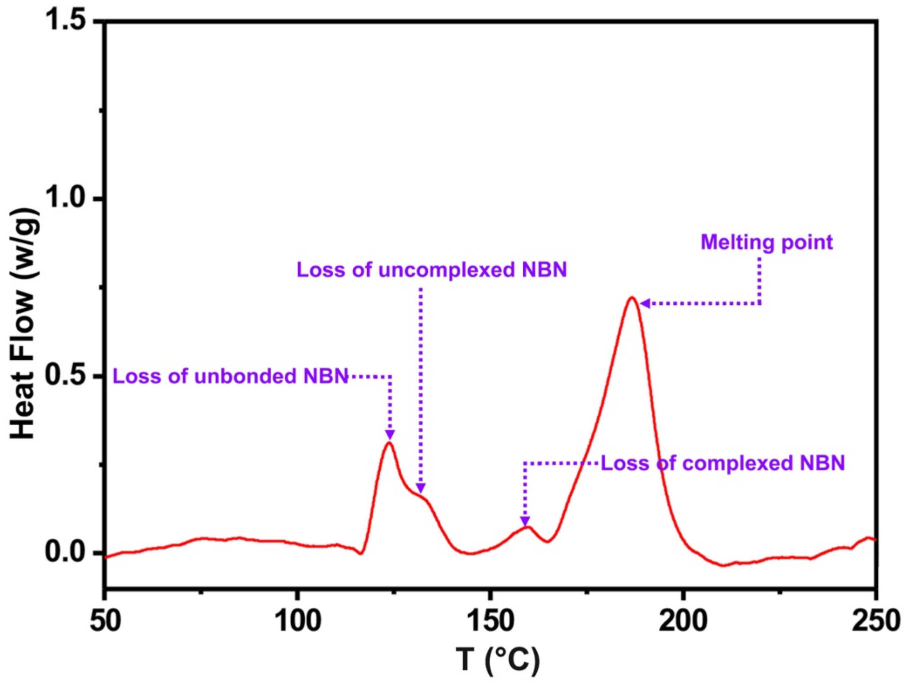

Supplementary Figure 20. DSC trace of EtP5-NBNα after exposure to 2-BPE vapor.

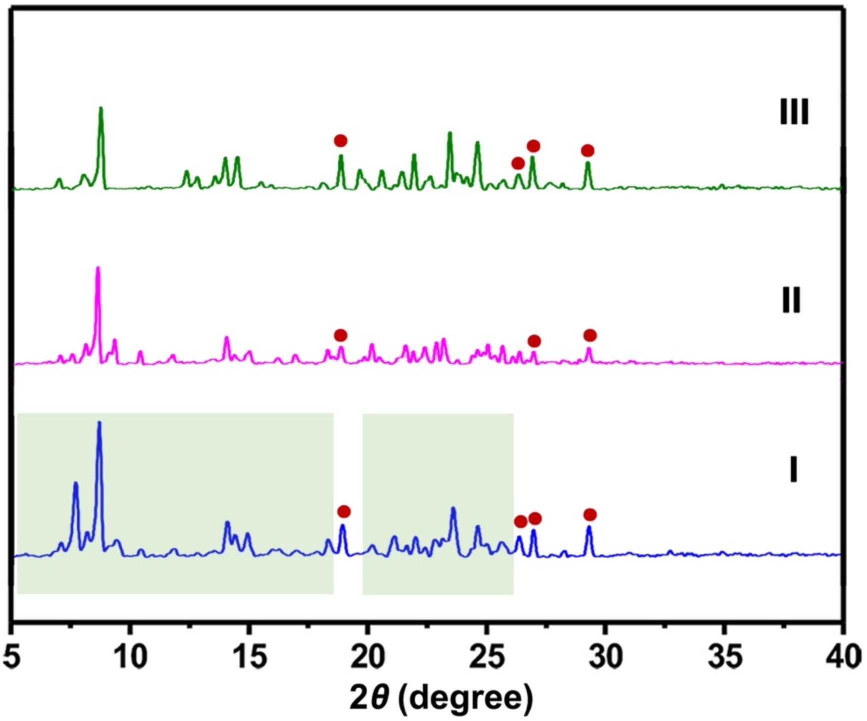

Supplementary Figure 21. PXRD patterns of EtP5-NBNα: (I) original EtP5-NBNα, after exposure to (II) 2-BBU vapor, and (III) 1-BBU vapor. The red dots represent the peaks of unbonded NBN crystalloids, and the green shades cover the peaks of the D-A complex.

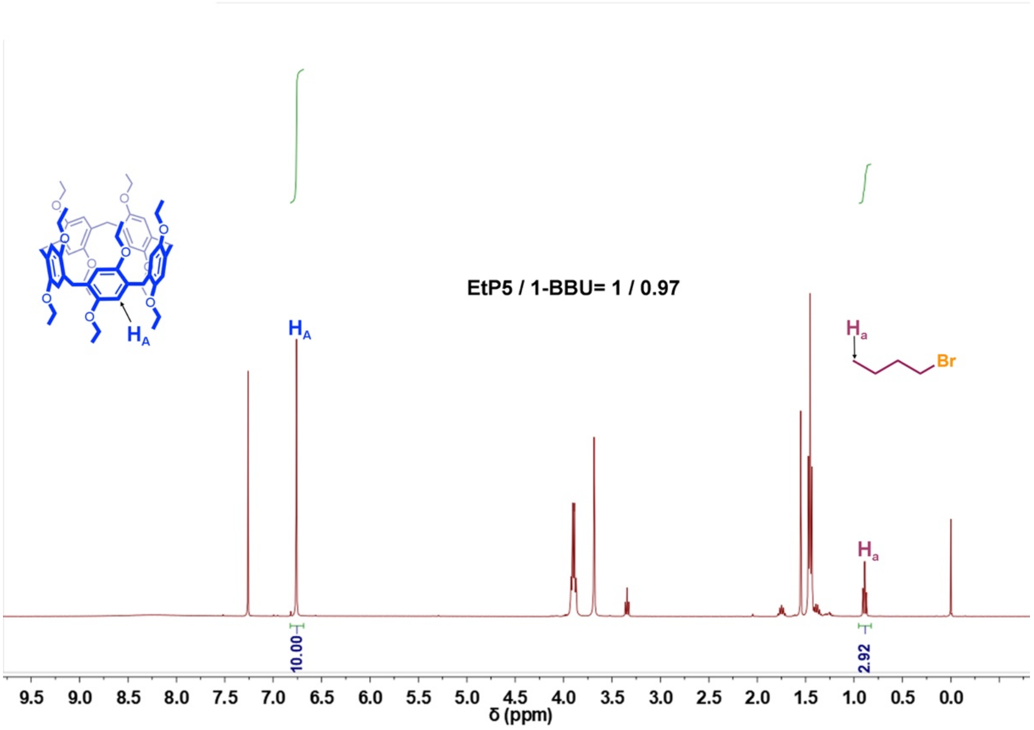

Supplementary Figure 22. ^{1}H NMR spectrum (400 MHz, $CDCl_3$, 298 K) of EtP5-DNBα after exposure to 1-BBU vapor.

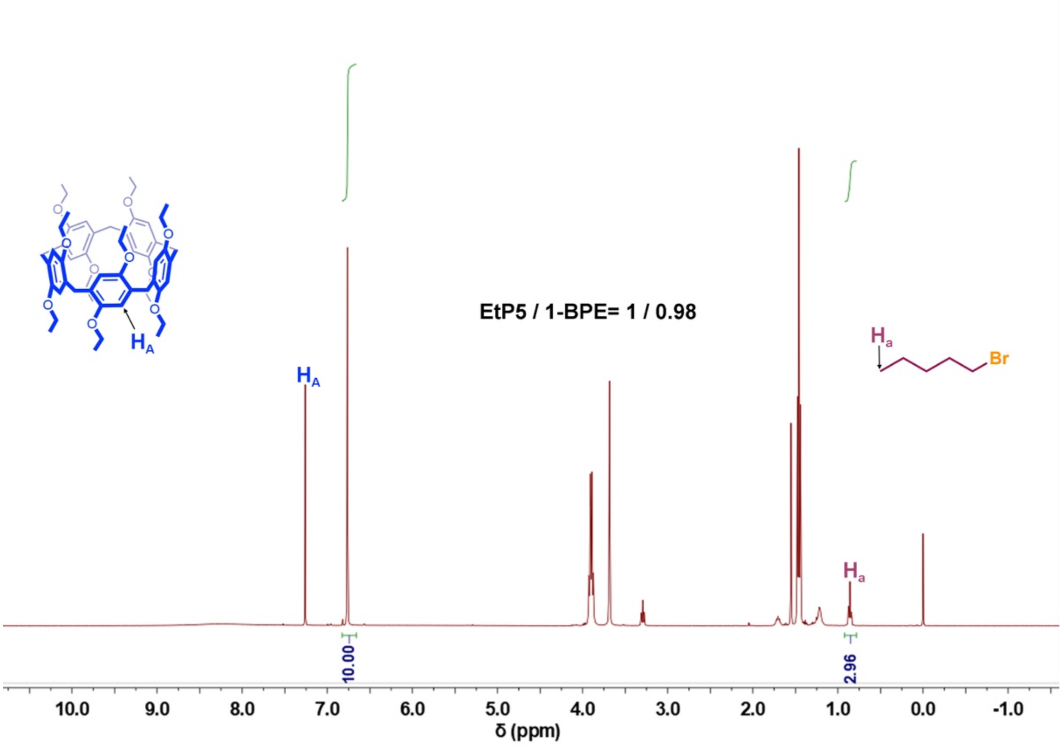

Supplementary Figure 23. ^{1}H NMR spectrum (400 MHz, $CDCl_3$, 298 K) of EtP5-DNBα after exposure to 1-BPE vapor.

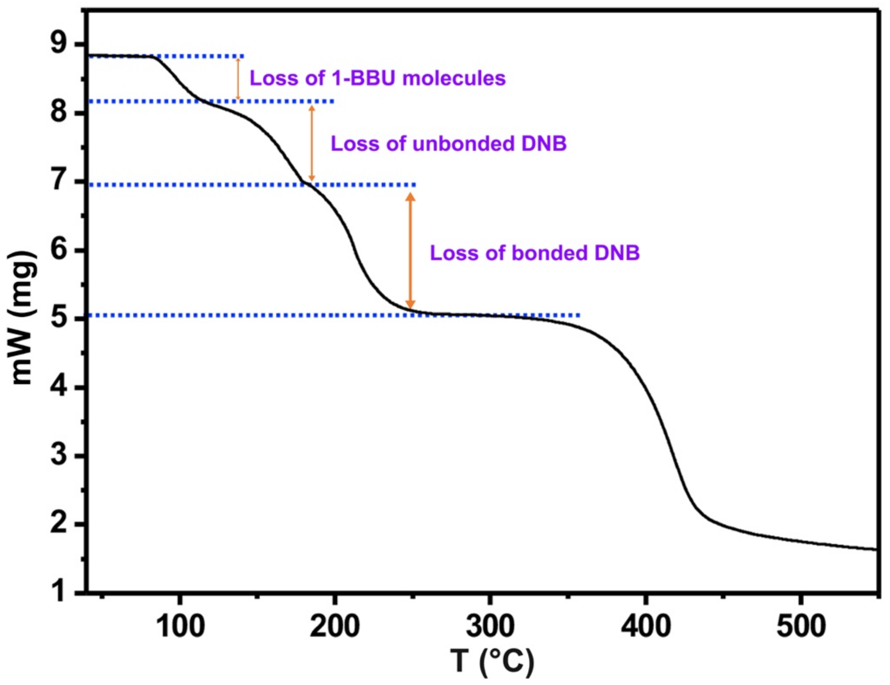

Supplementary Figure 24. TGA of EtP5-DNBα after exposure to 1-BBU vapor.

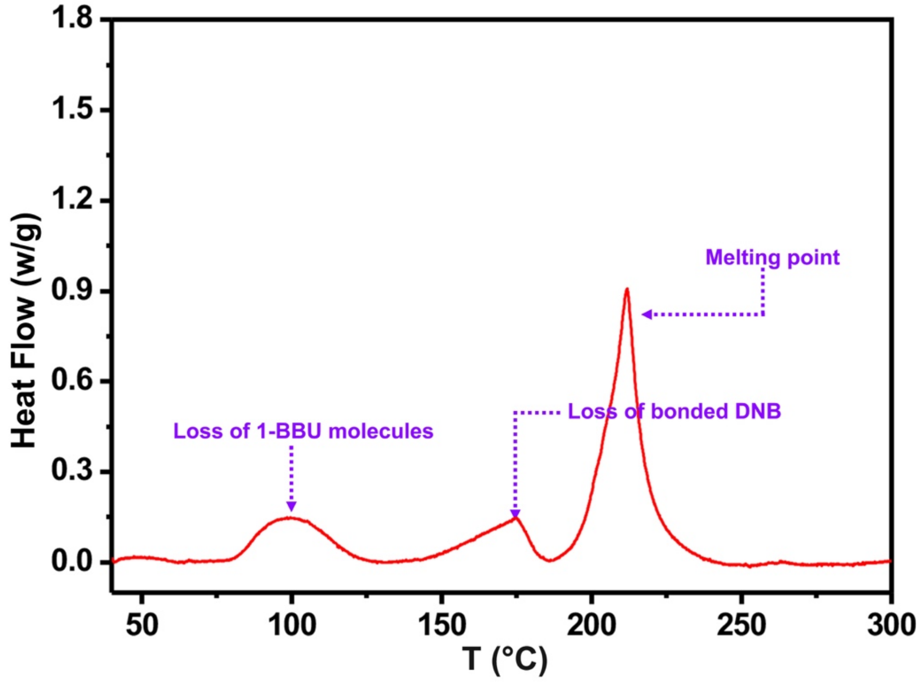

Supplementary Figure 25. DSC trace of EtP5-DNBα after exposure to 1-BBU vapor.

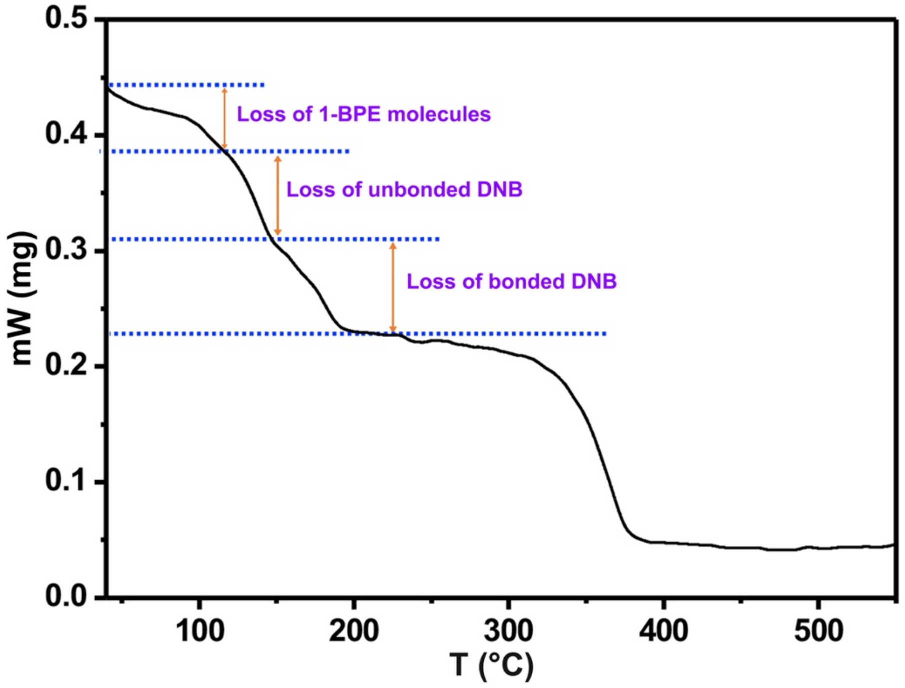

Supplementary Figure 26. TGA of EtP5-DNBα after exposure to 1-BPE vapor.

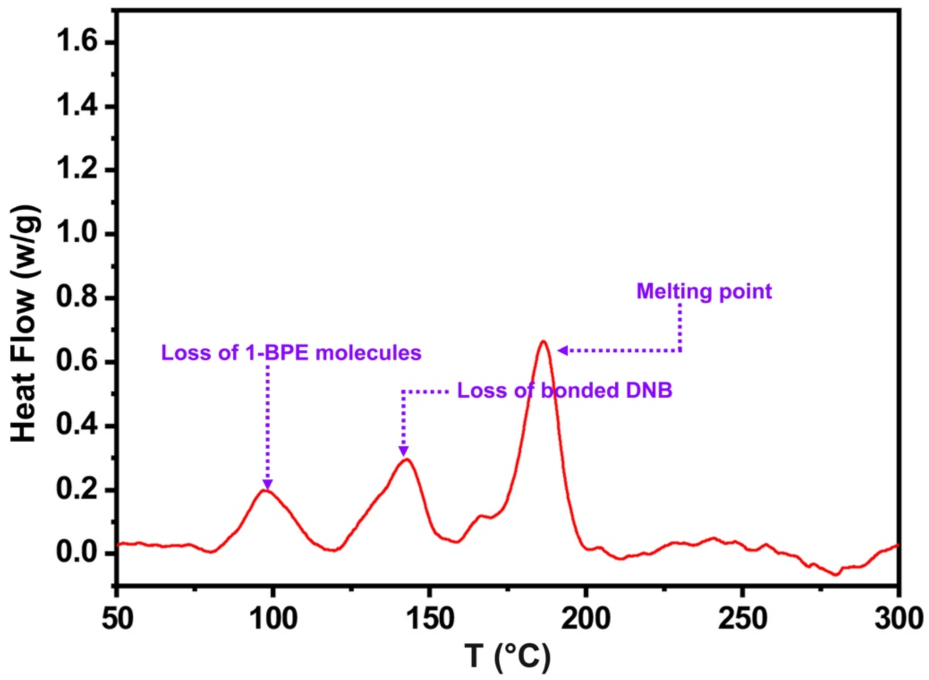

Supplementary Figure 27. DSC trace of EtP5-DNBα after exposure to 1-BPE vapor.

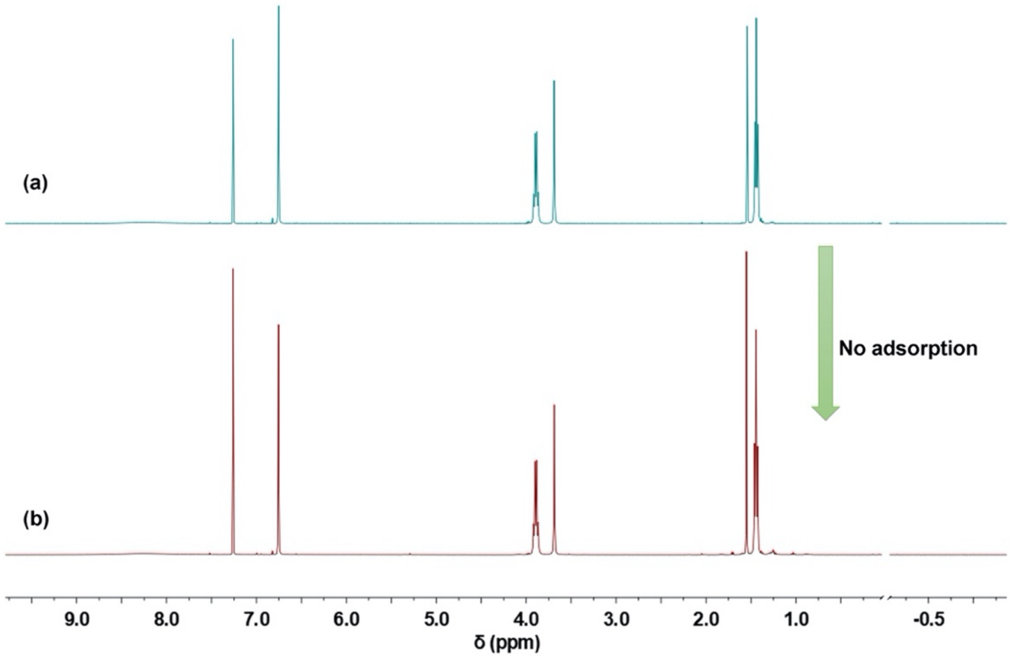

Supplementary Figure 28. ^{1}H NMR spectrum (400 MHz, $CDCl_3$, 298 K) of EtP5-DNBα (a) before and (b) after exposure to 2-BBU vapor.

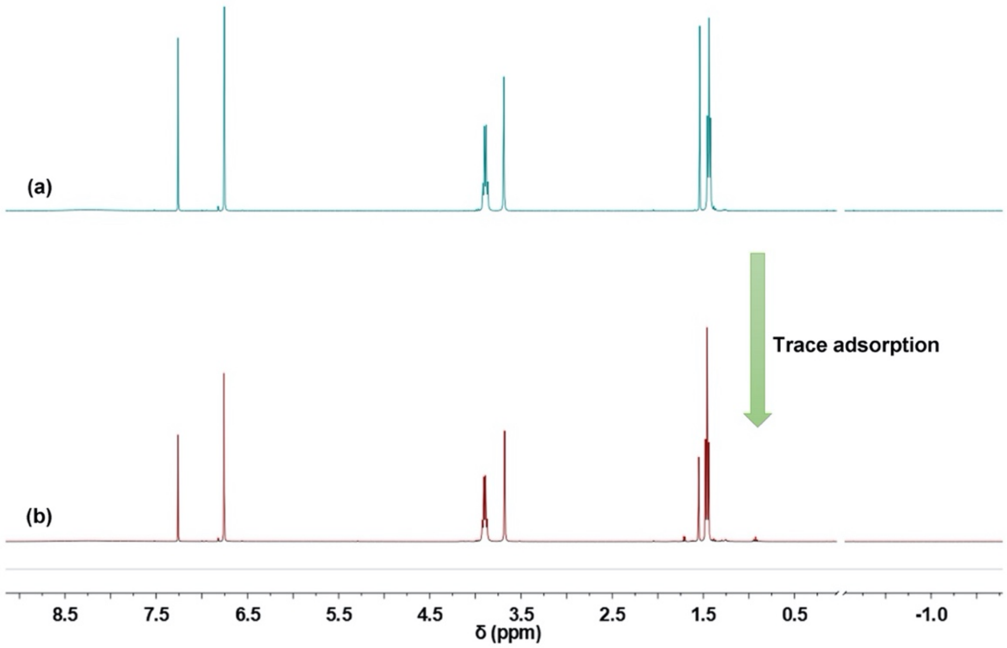

Supplementary Figure 29. ^{1}H NMR spectrum (400 MHz, $CDCl_3$, 298 K) of EtP5-DNBα (a) before and (b) after exposure to 2-BPE vapor.

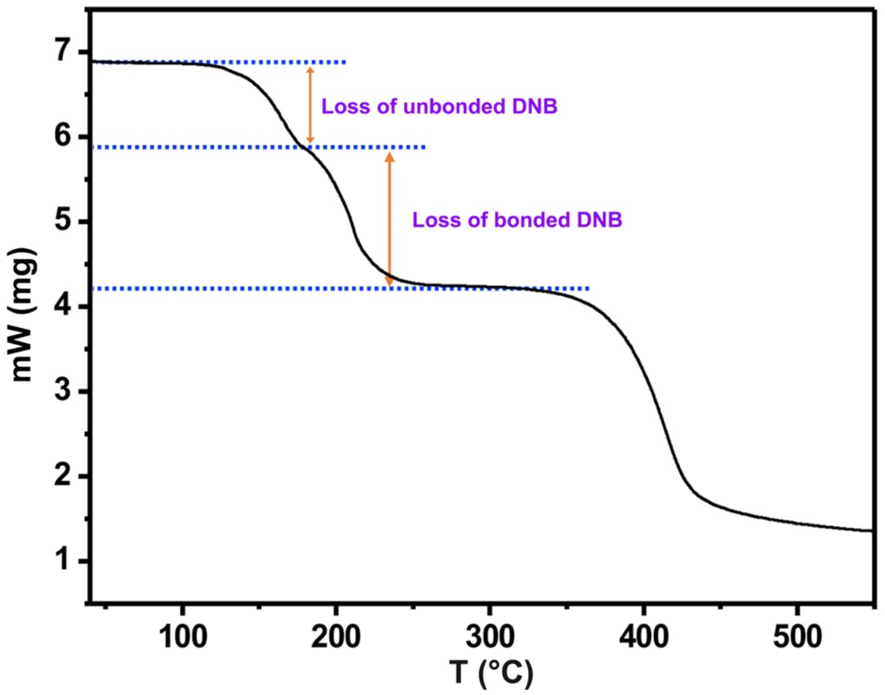

Supplementary Figure 30. TGA of EtP5-DNBα after exposure to 2-BBU vapor.

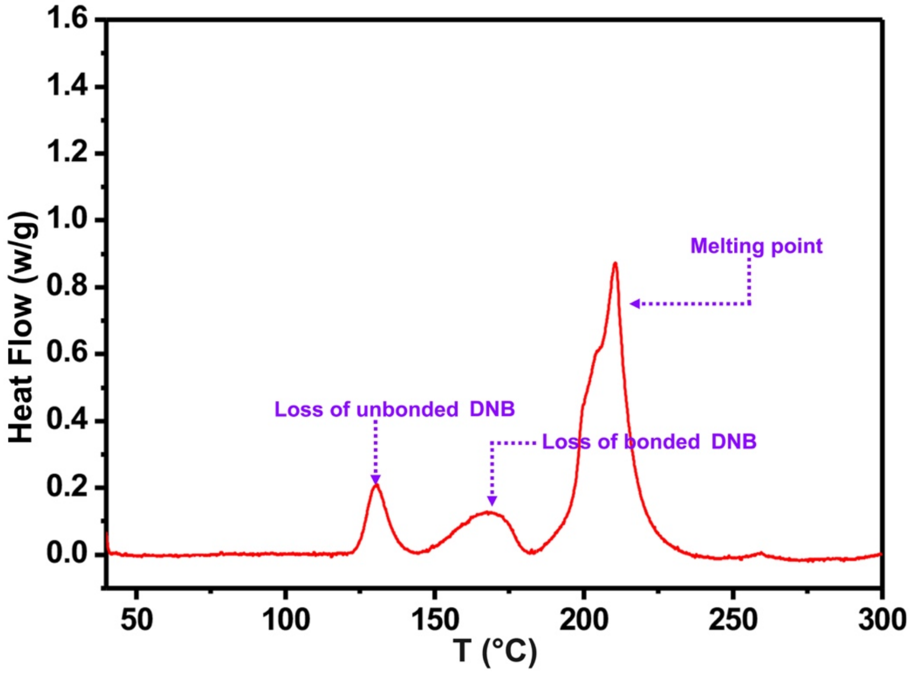

Supplementary Figure 31. DSC trace of EtP5-DNBα after exposure to 2-BBU vapor.

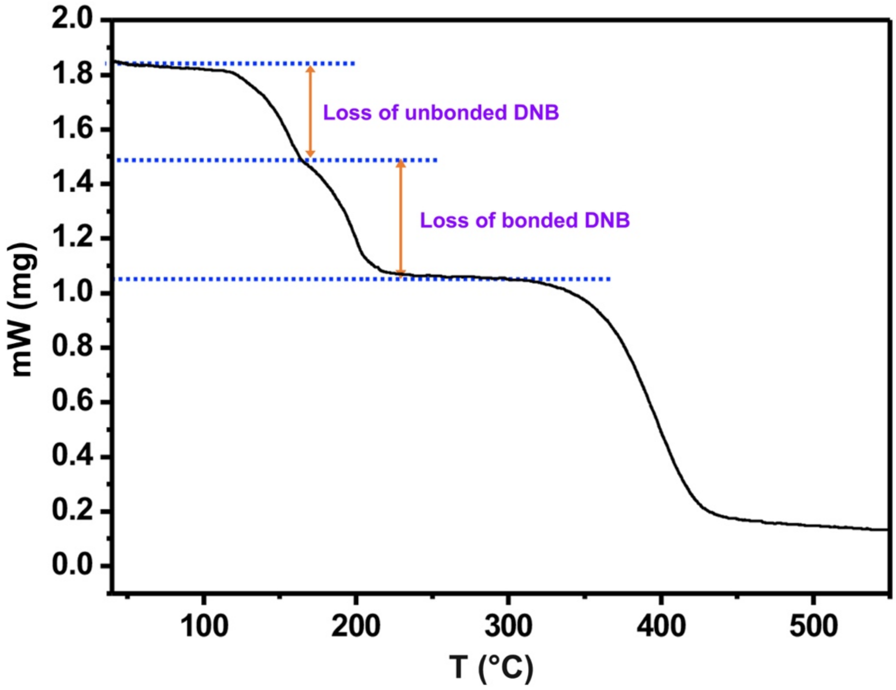

Supplementary Figure 32. TGA of EtP5-DNBα after exposure to 2-BPE vapor.

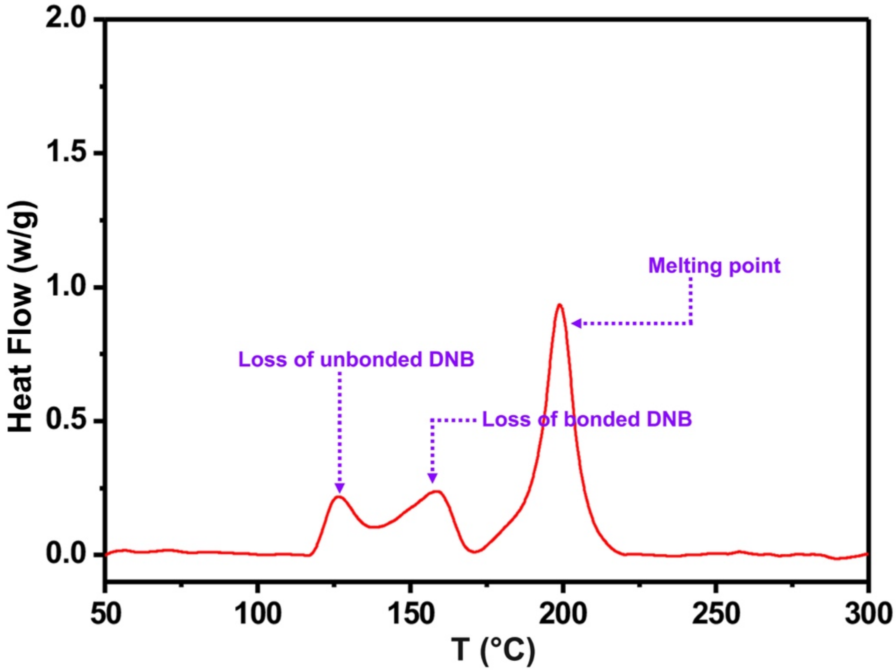

Supplementary Figure 33. DSC trace of EtP5-DNBα after exposure to 2-BPE vapor.

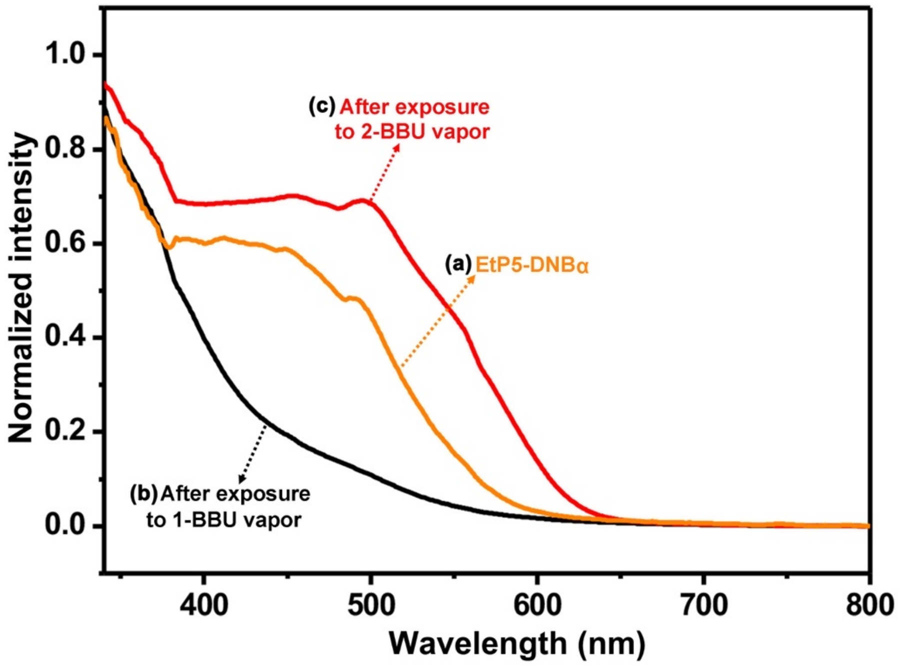

Supplementary Figure 34. Normalized solid-state UV/Vis absorption spectra: (a) original EtP5-DNBα, after exposure to (b) 1-BBU vapor, and (c) 2-BBU vapor.

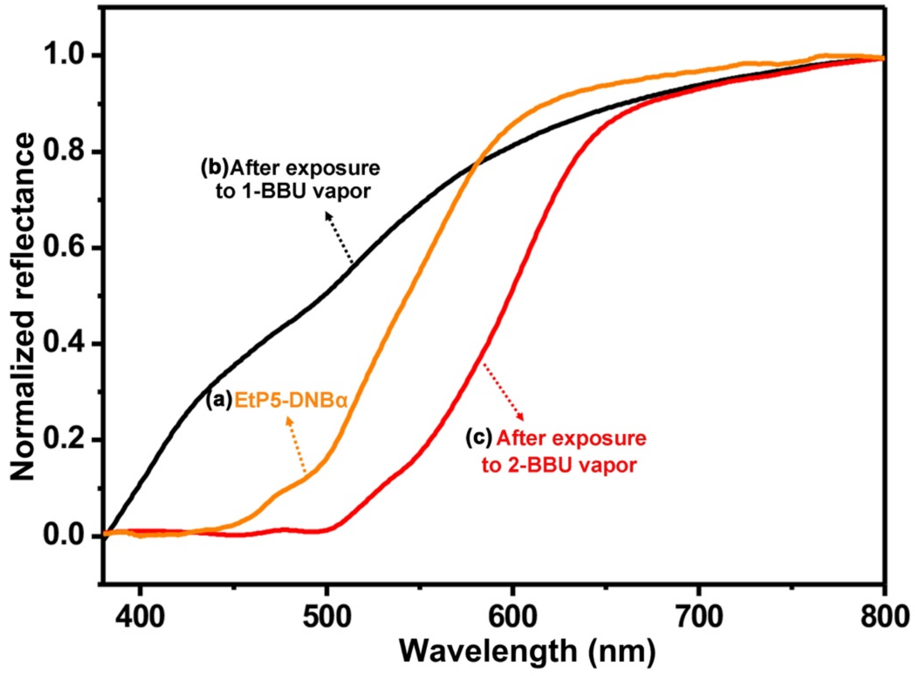

Supplementary Figure 35. Normalized solid-state UV/Vis diffuse reflection spectra: (a) original EtP5-NBNα, after exposure to (b) 1-BBU vapor, and (c) 2-BBU vapor.

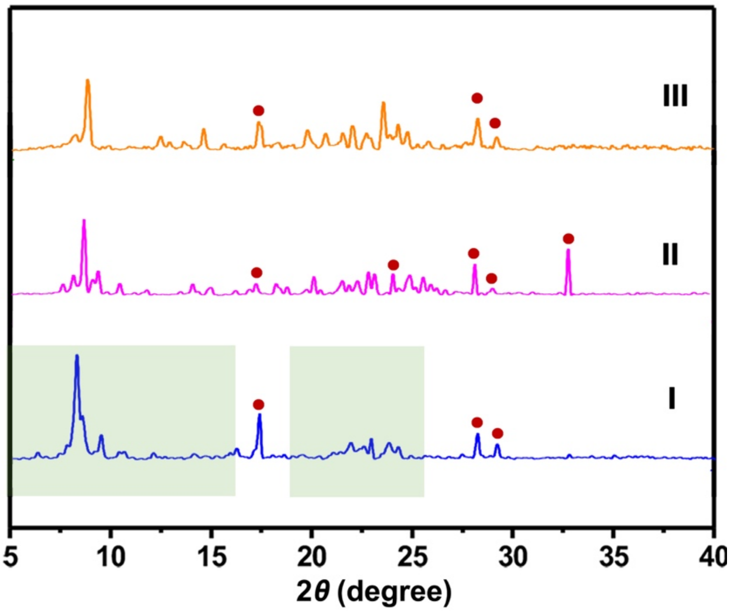

Supplementary Figure 36. PXRD patterns of EtP5-DNBα: (I) original EtP5-DNBα, after exposure to (II) 2-BBU vapor, and (III) 1-BBU vapor. The red dots represent the peaks of unbonded DNB crystalloids, and the green shades cover the peaks of the D-A complex.

2.3. X-Ray Crystallography

The hydrogen atoms were set in calculated positions and refined as riding atoms with a common fixed isotropic thermal parameter. NBN, 1-BBU and 1-BPE molecules in EtP5-NBNβ, 1-BBU⊂EtP5 and 1-BPE⊂EtP5 crystals are statistically positional disordered, respectively, and each set is independently occupied. Selected details of the data collection and structural refinement of each crystal can be found within Supplementary Tables 3 and 4 and full details are available in the corresponding CIF files. Crystallographic data (excluding structure factors) have been deposited with the Cambridge Crystallographic Data Centre and can be obtained free of charge via http://www.ccdc.cam.ac.uk/data_request/cif.

Supplementary Table 3. Single crystal data

Compound	EtP5-NBNβ	EtP5-DNBβ	EtP5-DNBγ
Crystallization Solvent	Methylcyclohexane/ CH_2Cl_2	Methylcyclohexane/ CH_2Cl_2	2-Bromopentane
Empirical formula	C_{55} H_{70} O_{10}, 1.5 (C_7 H_4 N_2 O_2)	C_{55} H_{70} O_{10}, C_6 H_4 N_2 O_4	C_{55} H_{70} O_{10}, 1.5 (C_6 H_4 N_2 O_4)
Formula weight	1113.29	1058.21	1143.28

Temperature / K	200.0	200.0	100.0
Crystal system	orthorhombic	monoclinic	orthorhombic
Space group	*Pbca*	*$P2_1/n$*	*Pbca*
a / Å	23.466(5)	12.4466 (11)	23.0860 (3)
b / Å	21.198(4)	21.358 (2)	21.1374 (3)
c / Å	24.629(5)	22.206 (3)	24.4170 (4)
α / °	90	90	90
β / °	90	92.814 (6)	90
γ / °	90	90	90
Volume / $Å^3$	12251 (4)	5896.1(11)	11915.0(3)
Z	8	118	51
ρ_{calc} g / cm^3	1.207	1.192	1.275
μ / mm^{-1}	0.436	0.441	0.091
Radiation	GaKα (λ = 1.34139)	GaKα (λ = 1.34139)	MoKα (λ = 0.71076)
F(000)	4752.0	2260.0	4872.0
2Θ range for data collection / °	3.277 to 57.190	2.499 to 57.056	1.8940 to 29.5520
Index ranges	-29 ≤ h ≤ 29, -26 ≤ k ≤ 24, -30 ≤ l ≤ 30	-15 ≤ h ≤ 15, -23 ≤ k ≤ 26, -27 ≤ l ≤ 27	-23 ≤ h ≤ 30, -26 ≤ k ≤ 28, -29 ≤ l ≤ 31
Reflections collected	121031	65888	78340
Independent reflections	12033 [R_{int} = 0.0464, R_{sigma} = 0.0254]	11557 [R_{int} = 0.0519, R_{sigma} = 0.0325]	14176 [R_{int} = 0.0290, R_{sigma} = 0.0264]
Goodness-of-fit on F^2	1.079	1.063	1.063
Final R indexes [I>= 2σ(I)]	R_1 = 0.0566, wR_2 = 0.1511	R_1 = 0.0867, wR_2 = 0.2549	R_1 = 0.0376, wR_2 = 0.0879
Final R indexes [all data]	R_1 = 0.0677, wR_2 = 0.1618	R_1 = 0.1159, wR_2 = 0.2885	R_1 = 0.0560, wR_2 = 0.0962
Largest diff. peak/hole/e $Å^{-3}$	0.775/-0.592	1.048/-0.562	0.301/-0.241
CCDC number	2247778	2247780	2247781

Supplementary Table 4. Single crystal data

Compound	1-BBU⊂EtP5	1-BPE⊂EtP5
Crystallization Solvent	1-Bromobutane	1-Bromopentane
Empirical formula	$C_4 H_9 Br, C_{55} H_{70} O_{10}$	$C_5 H_{11} Br, C_{55} H_{70} O_{10}$
Formula weight	1028.12	1042.15

Temperature / K	100.0	200.0
Crystal system	orthorhombic	orthorhombic
Space group	Pbcn	Pbcn
a / Å	43.2435(6)	42.6400(17)
b / Å	15.5584(2)	15.8537(6)
c / Å	16.0281(2)	16.7412(7)
α / °	90	90
β / °	90	90
γ / °	90	90
Volume / $Å^3$	10783.7(2)	11317.1(8)
Z	47	8
ρ_{calc} g / cm^3	1.267	1.223
μ / mm^{-1}	1.498	0.965
Radiation	CuKα (λ = 1.54184)	GaKα (λ = 1.34139)
F(000)	4384.0	4448.0
2Θ range for data collection / °	2.043 to 75.901	2.587 to 57.445
Index ranges	-54 ≤ h ≤ 53, -19 ≤ k ≤ 19, -18 ≤ l ≤ 19	-53 ≤ h ≤ 53, -19 ≤ k ≤ 11, -19 ≤ l ≤ 21
Reflections collected	56184	129093
Independent reflections	10940 [R_{int} = 0.0409, R_{sigma} = 0.0278]	11661 [R_{int} = 0.0583, R_{sigma} = 0.0286]
Goodness-of-fit on F^2	1.024	1.056
Final R indexes [I>= 2σ(I)]	R_1 = 0.0722, wR_2 = 0.1915	R_1 = 0.0780, wR_2 = 0.2169
Final R indexes [all data]	R_1 = 0.0795, wR_2 = 0.1990	R_1 = 0.0922, wR_2 = 0.2277
Largest diff. peak/hole/e $Å^{-3}$	1.683/-1.536	1.007/-0.686
CCDC number	2247790	2247792

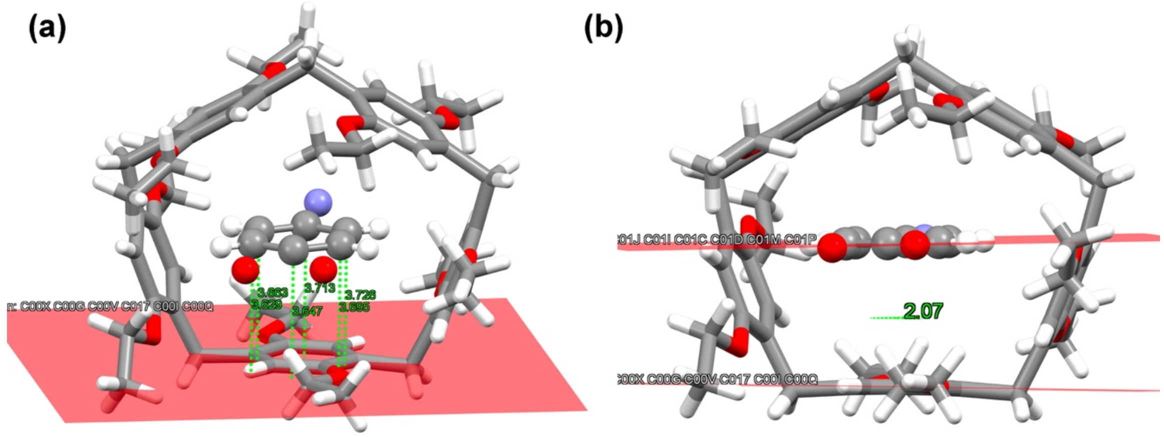

Supplementary Figure 37. Illustration of (a) π···π interaction and (b) dihedral angle value between the included NBN and EtP5 in EtP5-NBNβ.

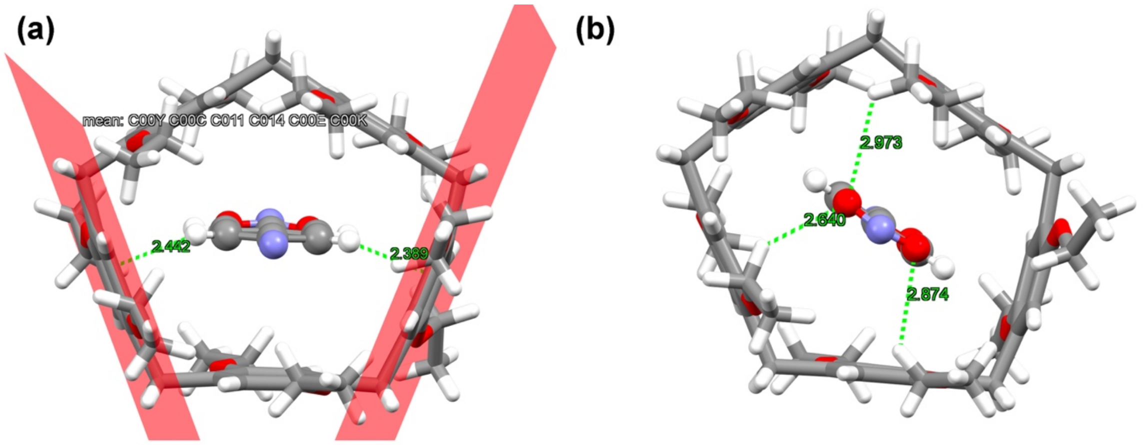

Supplementary Figure 38. Illustration of (a) C−H···π and (b) C−H···O interactions between the included NBN and EtP5 in EtP5-NBNβ.

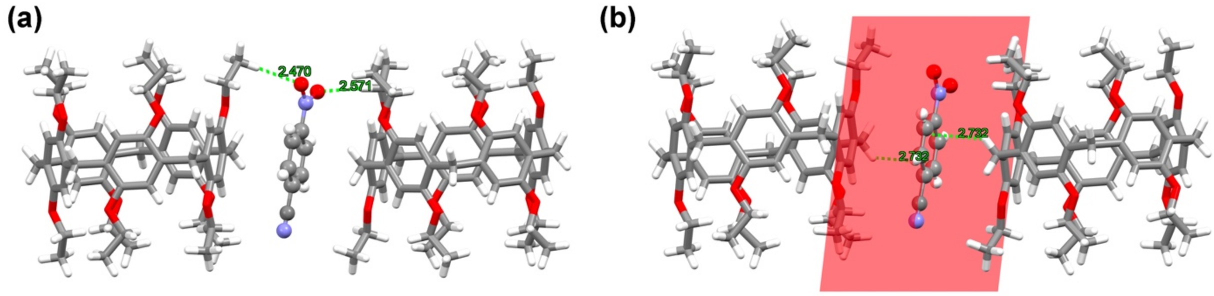

Supplementary Figure 39. Illustration of (a) C−H···O and (b) C−H···π interactions between the uncomplexed NBN and EtP5 in the structure of EtP5-NBNβ.

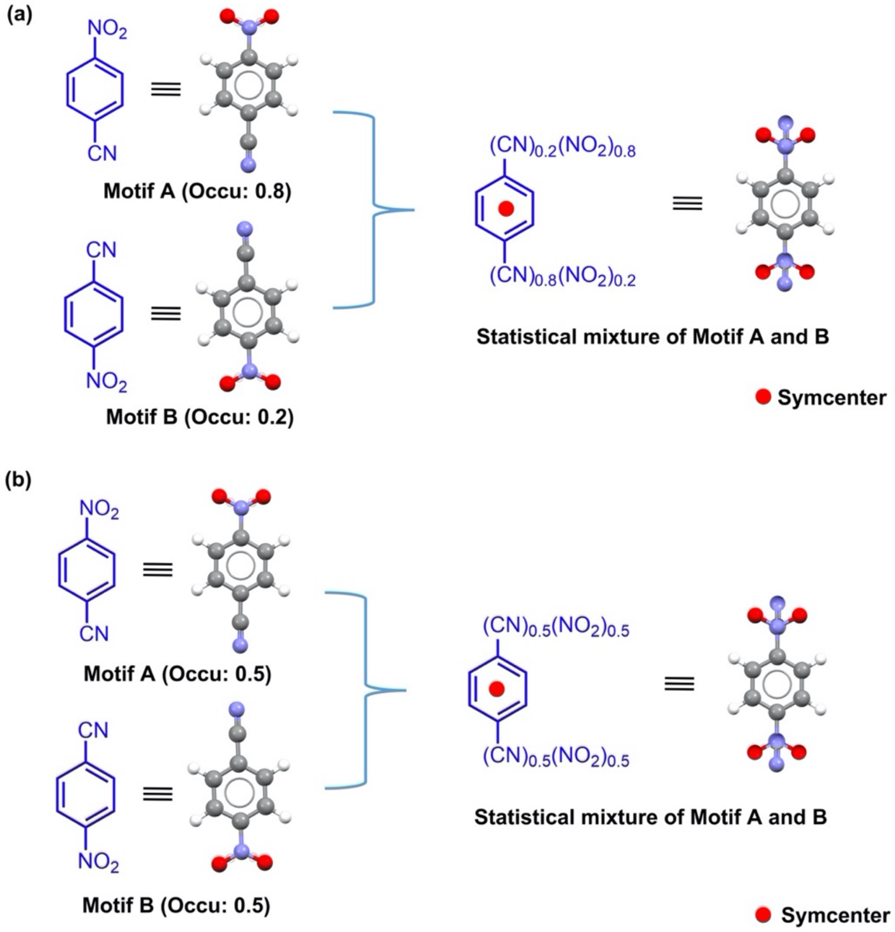

Supplementary Figure 40. Schematic representation of the statistically positional disorder of (a) complexed and (b) uncomplexed NBN in the crystal structure of EtP5-NBNβ.

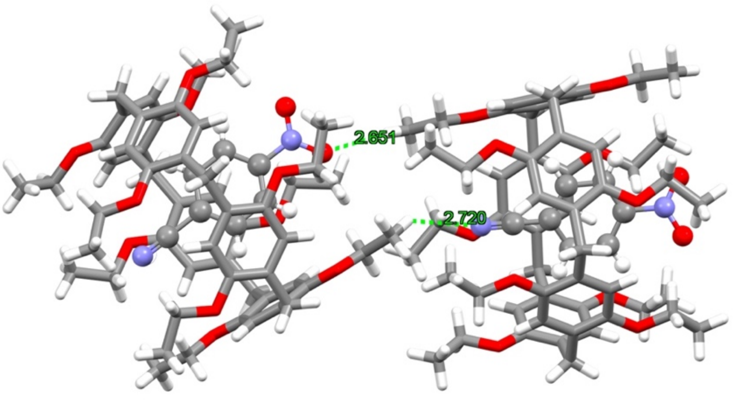

Supplementary Figure 41. Illustration of C−H···O and C−H···N interactions between the terminal atoms of the included NBN and the adjacent EtP5 in the structure of EtP5-NBNβ.

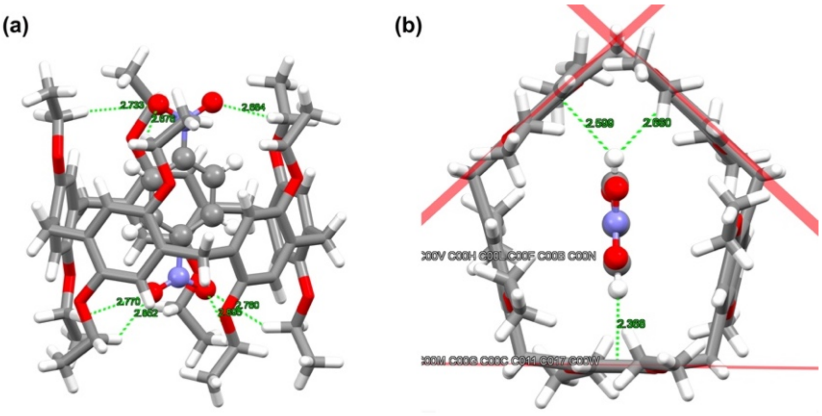

Supplementary Figure 42. Illustration of (a) C−H···O and (b) C−H···π interactions between DNB and EtP5 in the structure of EtP5-DNBβ.

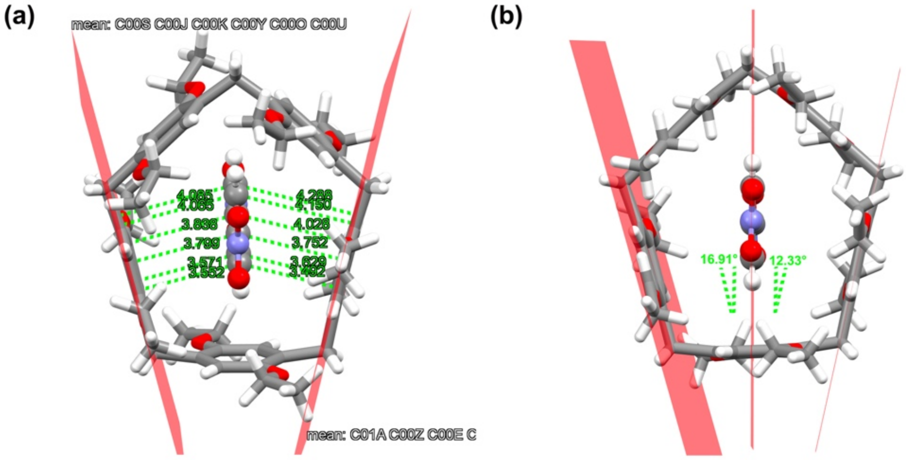

Supplementary Figure 43. Illustration of (a) π···π interactions and (b) dihedral angle values between DNB and EtP5 in the structure of EtP5-DNBβ.

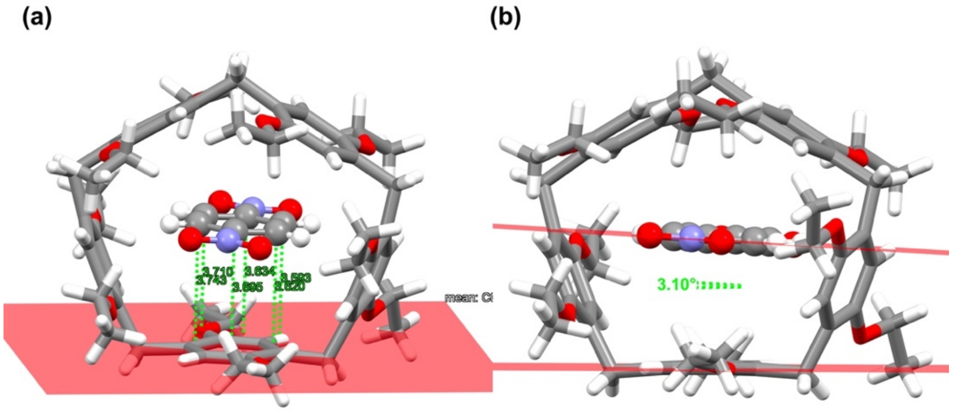

Supplementary Figure 44. Illustration of (a) π···π interaction and (b) dihedral angle value between the included DNB and EtP5 in the structure of EtP5-DNBγ.

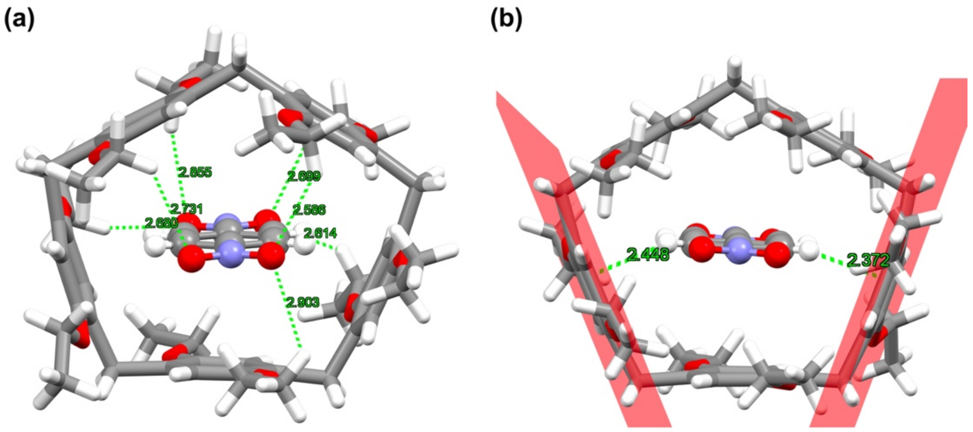

Supplementary Figure 45. Illustration of (a) C−H···O and (b) C−H···π interactions between the included DNB and EtP5 in the structure of EtP5-DNBγ.

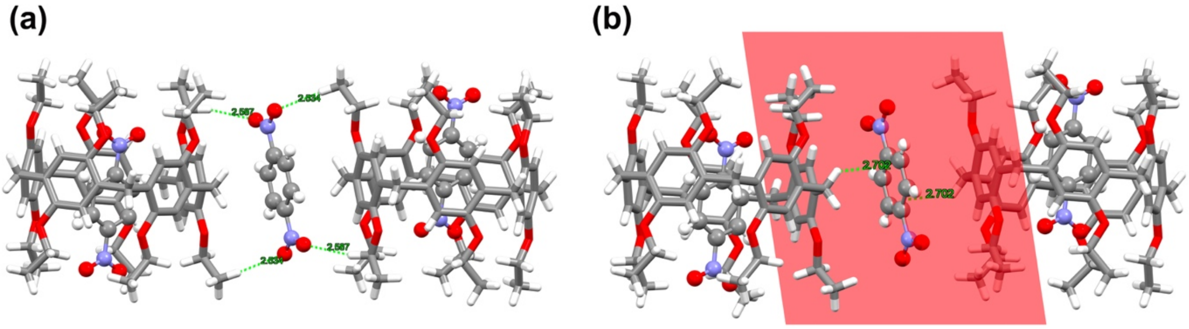

Supplementary Figure 46. Illustration of (a) C−H···O and (b) C−H···π interactions between the uncomplexed DNB and EtP5 in the structure of EtP5-DNBγ.

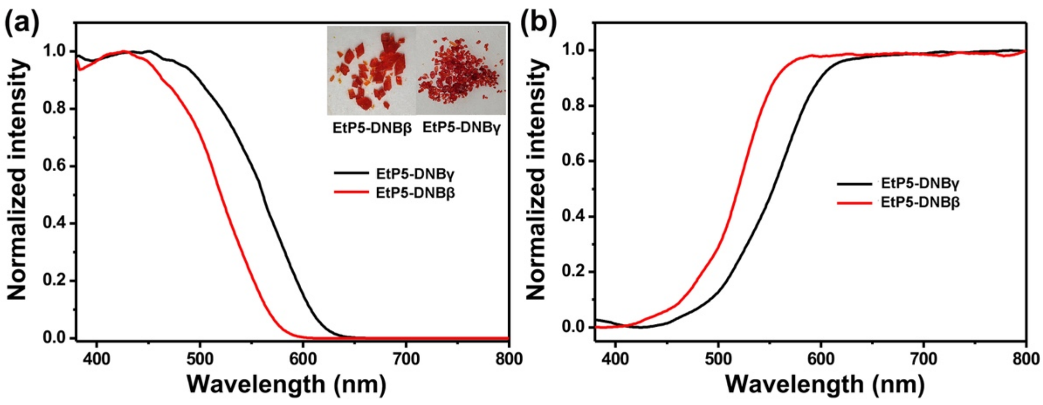

Supplementary Figure 47. Comparison of (a) solid-state UV/Vis absorption spectra and (b) diffuse reflection spectra between the crystals of EtP5-DNBβ (red line) and EtP5-DNBγ (black line).

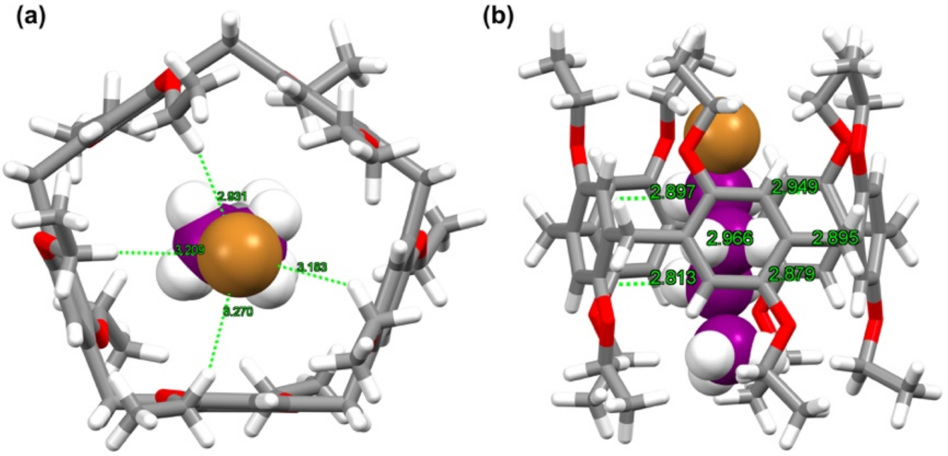

Supplementary Figure 48. Illustration of (a) C−H···Br and (b) C−H···π interactions between 1-BBU and EtP5 in the structure of 1-BBU⊂EtP5.

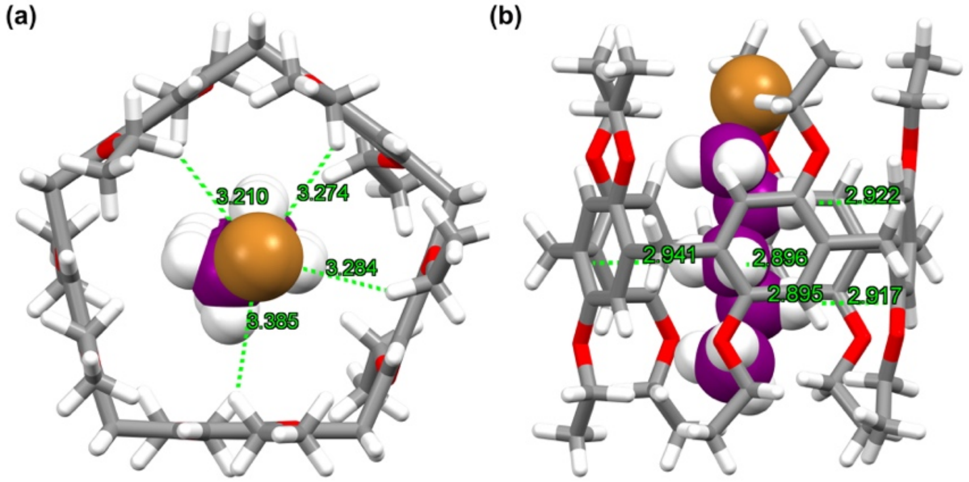

Supplementary Figure 49. Illustration of (a) C−H···Br and (b) C−H···π interactions between 1-BPE and EtP5 in the structure of 1-BPE⊂EtP5.

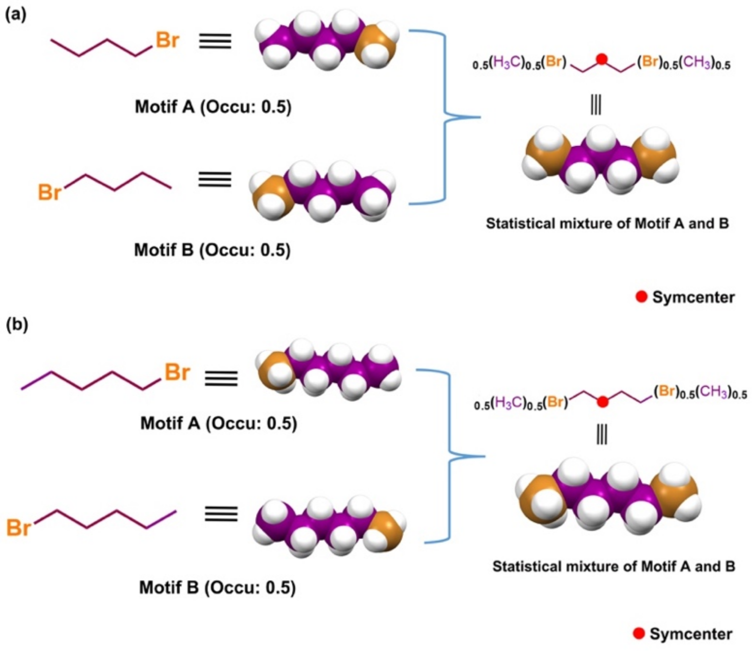

Supplementary Figure 50. Schematic representation of the statistically positional disorder of (a) 1-BBU and (b) 1-BPE in the crystal structure of 1-BBU⊂EtP5 and 1-BPE⊂EtP5, respectively.

2.4. Mechanism Study of the On-off Type Vapochromic Behavior

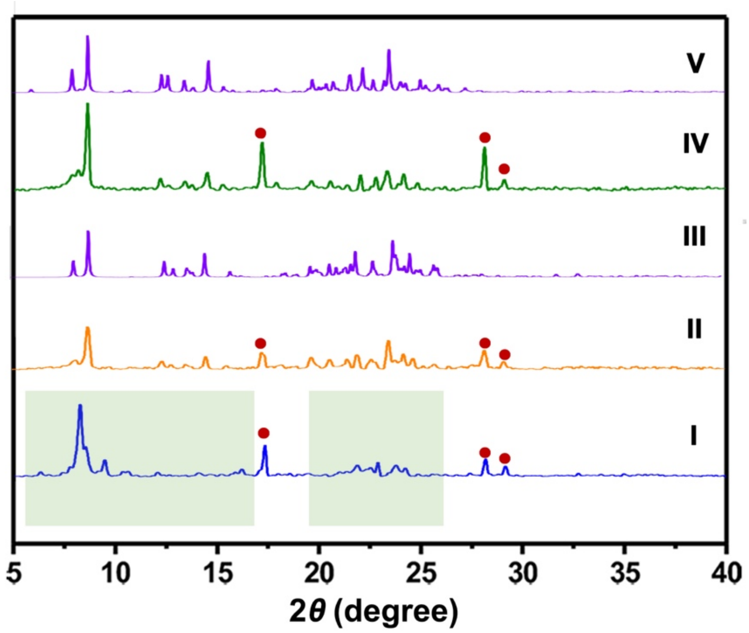

Supplementary Figure 51. PXRD patterns of EtP5-DNBα: (I) original EtP5-DNBα; (II) after exposure to 1-BBU vapor; (III) simulated from the crystal structure of 1-BBU⊂EtP5; (IV) after exposure to 1-BPE vapor; (V) simulated from the crystal structure of 1-BPE⊂EtP5. The red dots represent the peaks of unbonded DNB crystalloids, and the green shades cover the peaks of the D-A complex.

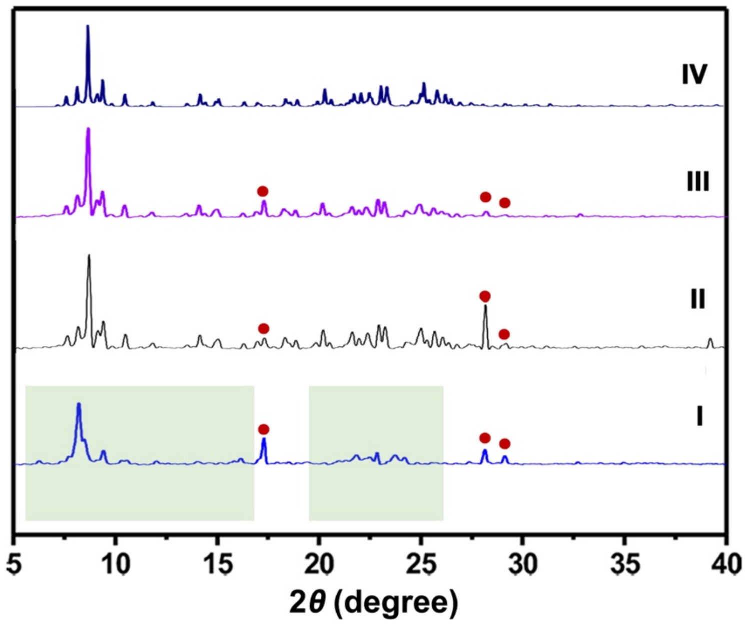

Supplementary Figure 52. PXRD patterns of EtP5-DNBα: (I) original EtP5-DNBα; (II) after exposure to 2-BBU vapor; (III) after exposure to 2-BPE vapor; (IV) simulated from the crystal structure of EtP5-DNBγ. The red dots represent the peaks of unbonded DNB crystalloids. The red dots represent the peaks of unbonded DNB crystalloids, and the green shades cover the peaks of the D-A complex.

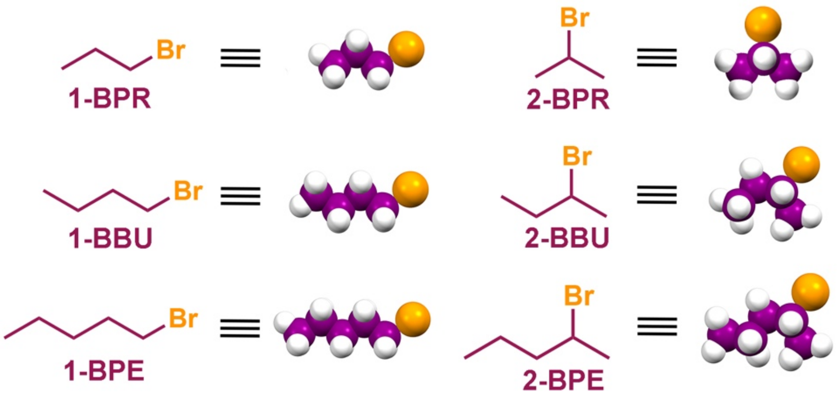

Supplementary Figure 53. Chemical structures and cartoon representations of 1-BPR, 2-BPR, 1-BBU, 2-BBU, 1-BPE, and 2-BPE with different alkyl chain lengths.

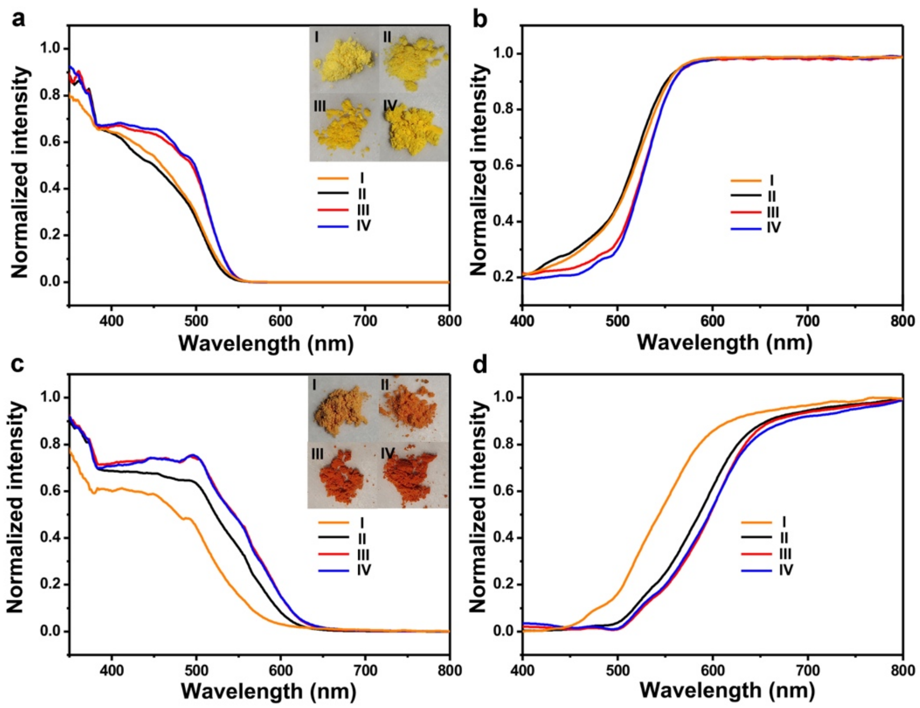

Supplementary Figure 54. (a) Normalized solid-state UV−vis absorption spectra: (I) original EtP5-NBNα, after exposure to (II) 1-BPR vapor, (III) 2-BPR vapor and (IV) 1-BPR/2-BPR mixture vapor. (b) Normalized diffuse reflectance spectra: (I) original EtP5-NBNα, after exposure to (II) 1-BPR vapor, (III) 2-BPR vapor and (IV) 1-BPR/2-BPR mixture vapor. (c) Normalized solid-state UV−vis absorption spectra: (I) original EtP5-DNBα, after exposure to (II) 1-BPR vapor, (III) 2-BPR vapor and (IV) 1-BPR/2-BPR mixture vapor. (d) Normalized diffuse reflectance spectra: (I) original EtP5-DNBα, after exposure to (II) 1-BPR vapor, (III) 2-BPR vapor and (IV) 1-BPR/2-BPR mixture vapor.

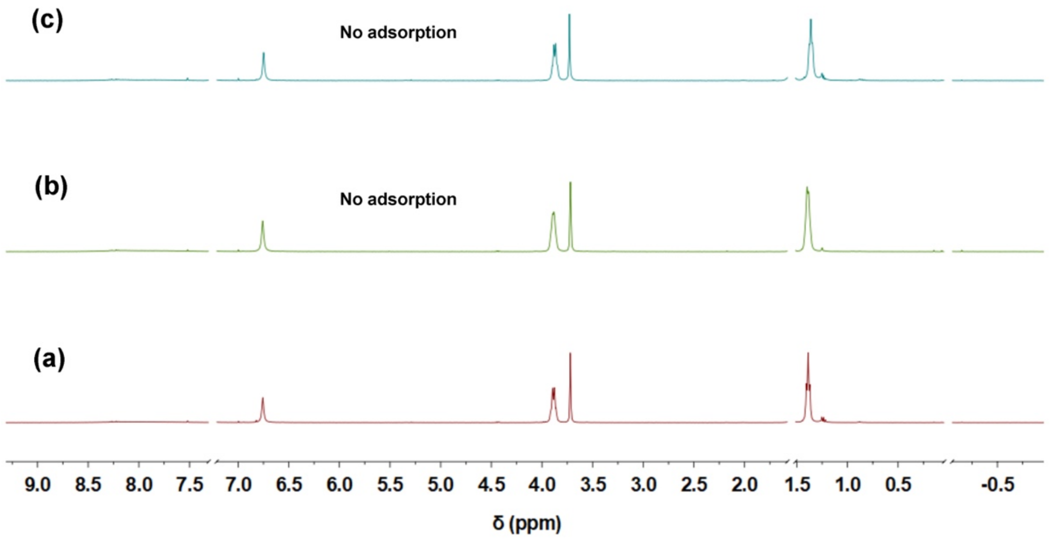

Supplementary Figure 55. ^{1}H NMR spectra (400 MHz, $CDCl_3$, 298 K) of EtP5-NBNα: (a) original EtP5-NBNα; (b) after exposure to 1-BPR vapor; (c) after exposure to 2-BPR vapor.

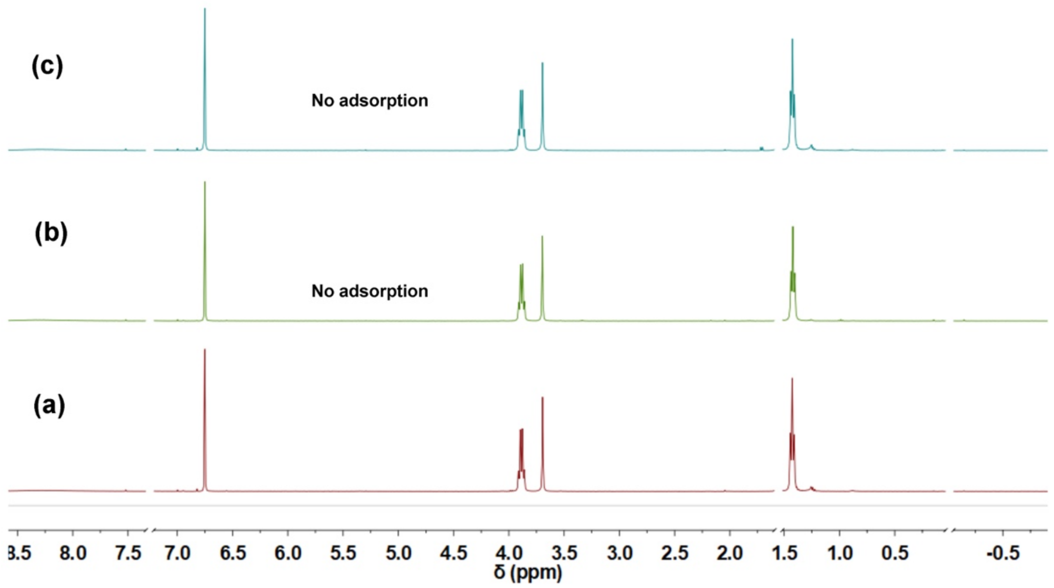

Supplementary Figure 56. ^{1}H NMR spectra (400 MHz, $CDCl_3$, 298 K) of EtP5-DNBα: (a) original EtP5-DNBα; (b) after exposure to 1-BPR vapor; (c) after exposure to 2-BPR vapor.

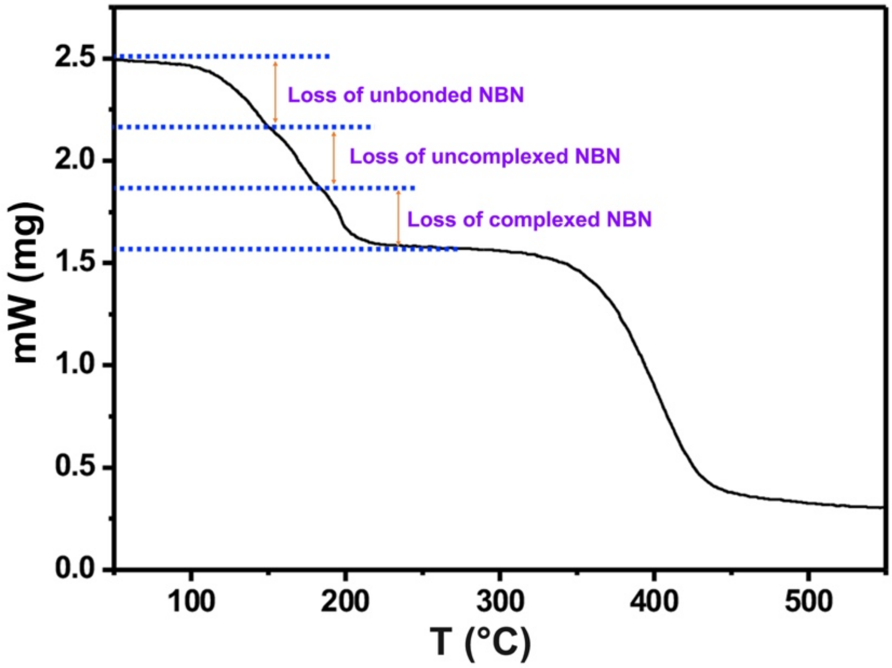

Supplementary Figure 57. TGA of EtP5-NBNα after exposure to 1-BPR vapor.

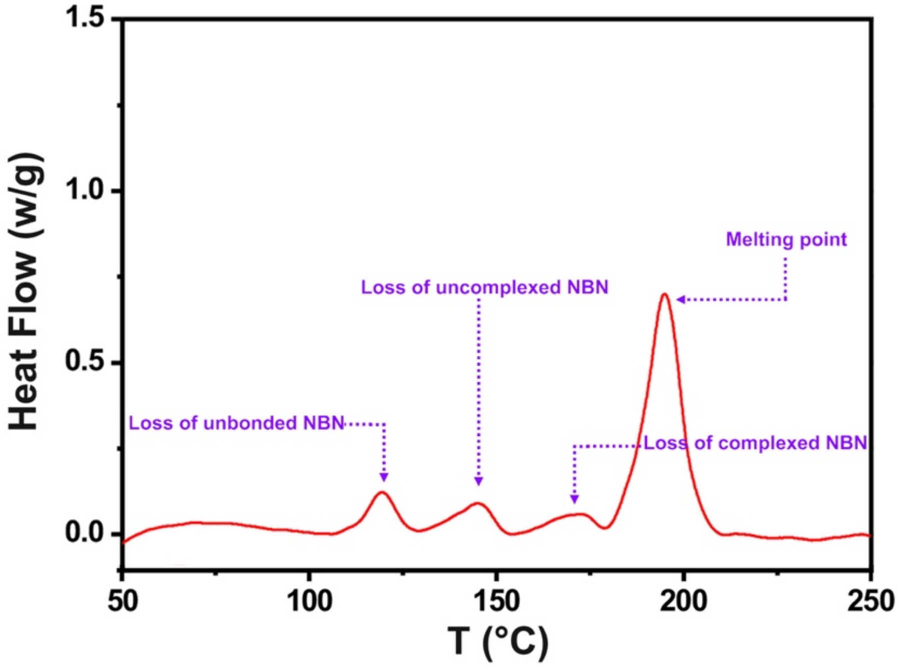

Supplementary Figure 58. DSC trace of EtP5-NBNα after exposure to 1-BPR vapor.

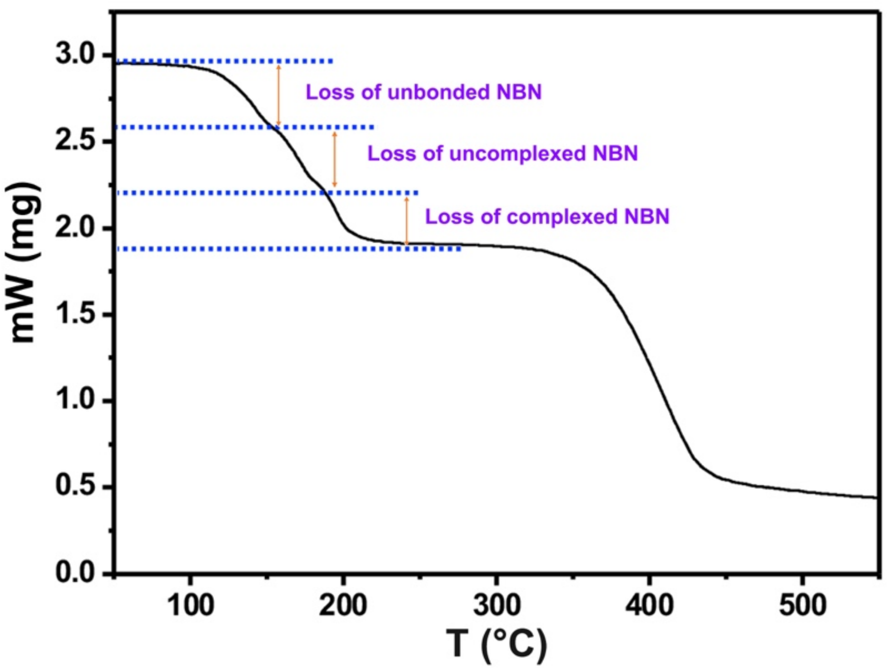

Supplementary Figure 59. TGA of EtP5-NBNα after exposure to 2-BPR vapor.

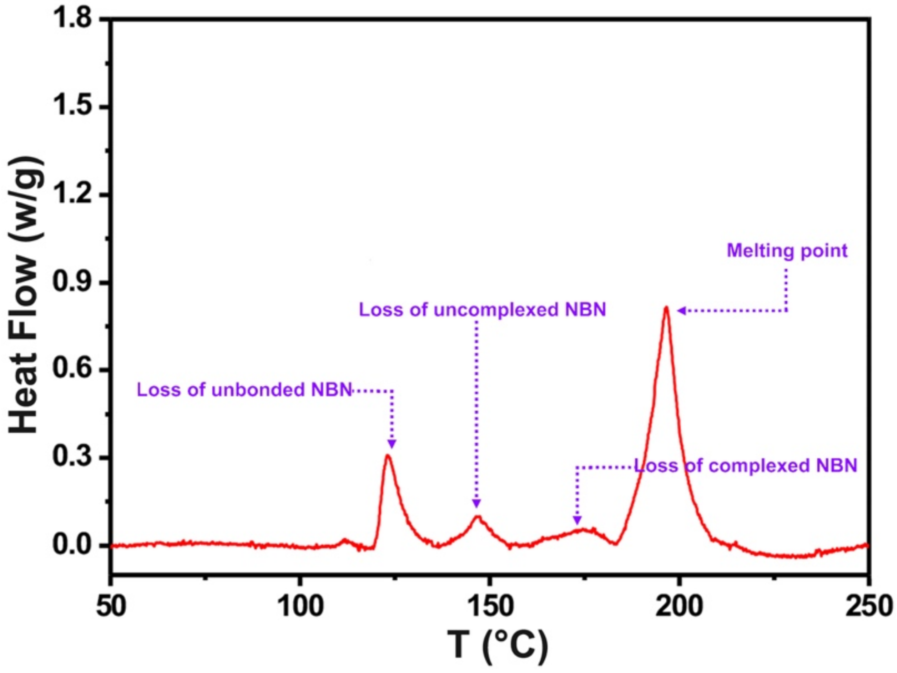

Supplementary Figure 60. DSC trace of EtP5-NBNα after exposure to 2-BPR vapor.

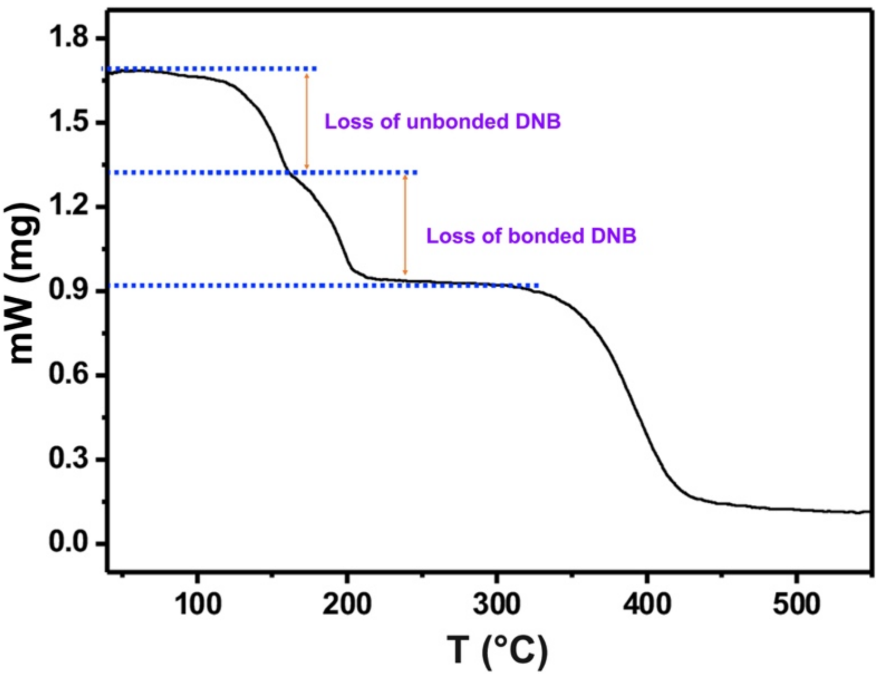

Supplementary Figure 61. TGA of EtP5-DNBα after exposure to 1-BPR vapor.

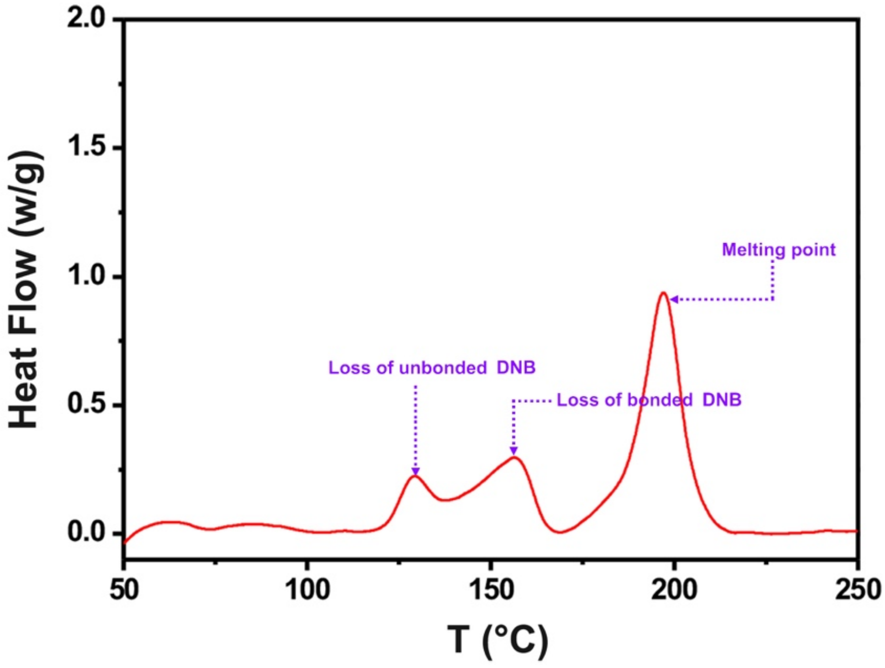

Supplementary Figure 62. DSC trace of EtP5-DNBα after exposure to 1-BPR vapor.

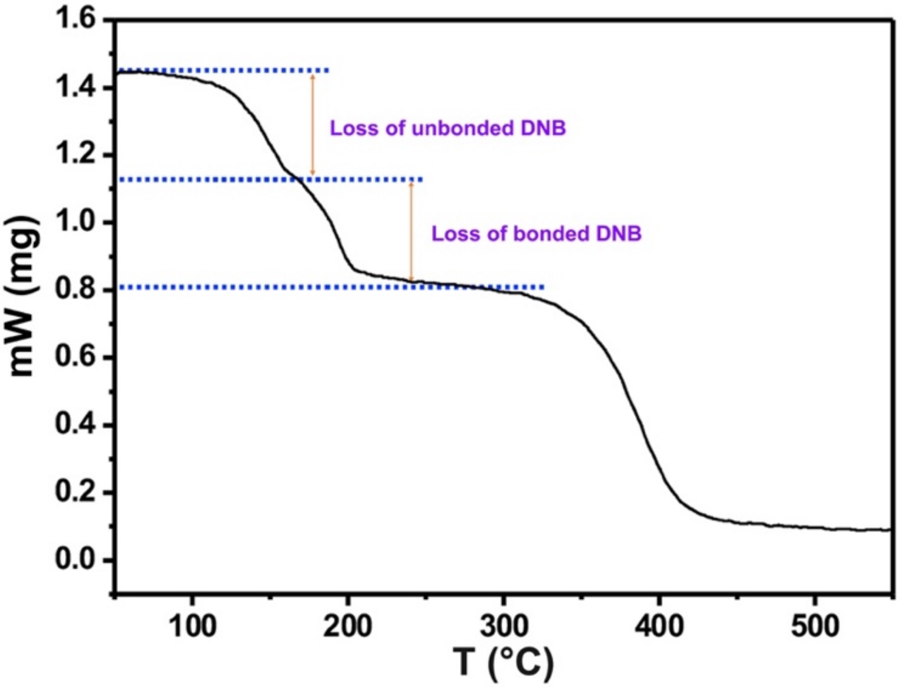

Supplementary Figure 63. TGA of EtP5-DNBα after exposure to 2-BPR vapor.

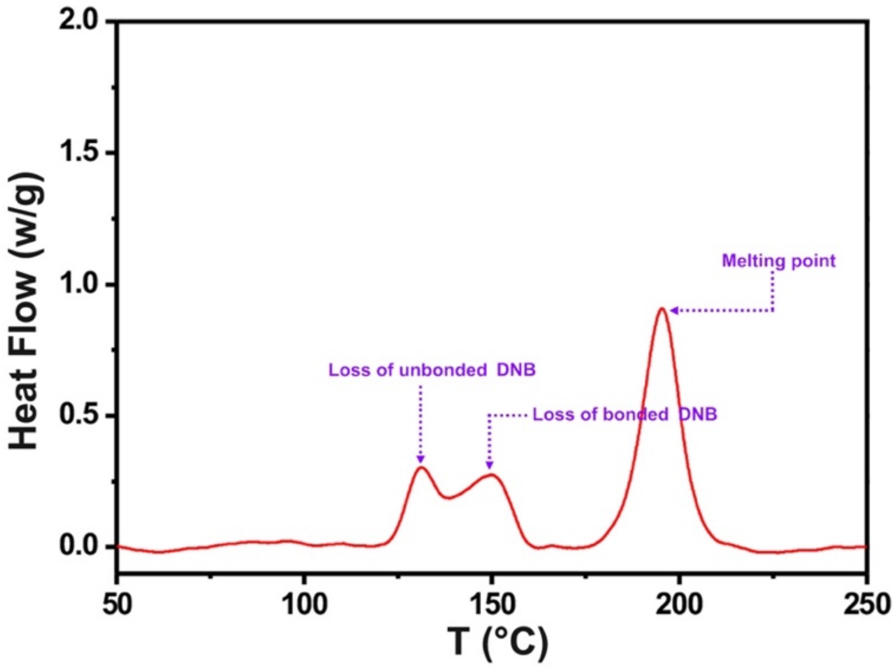

Supplementary Figure 64. DSC trace of EtP5-DNBα after exposure to 2-BPR vapor.

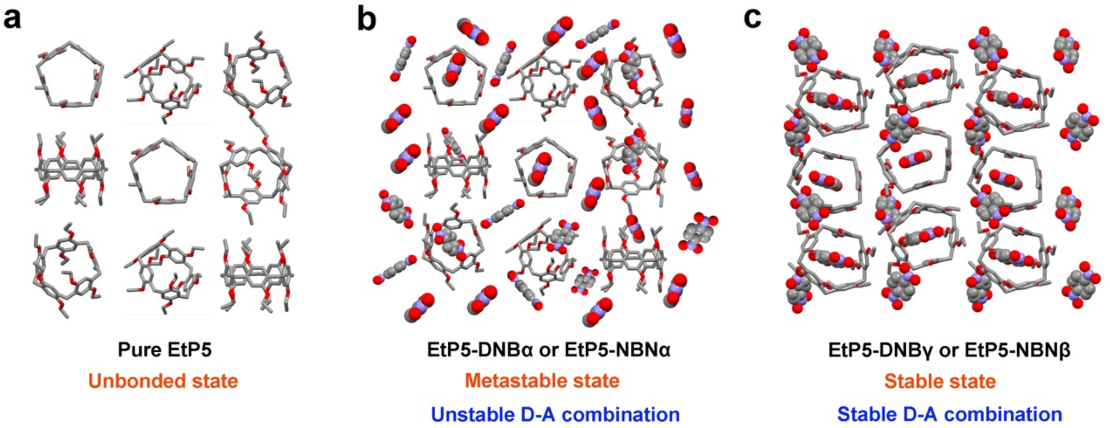

Supplementary Figure 65. Solid-state EtP5 with different binding states with the acceptors: (a) pure EtP5; (b) EtP5-DNBα or EtP5-NBNα (grinding state); (c) EtP5-DNBγ or EtP5-NBNβ (crystal state).

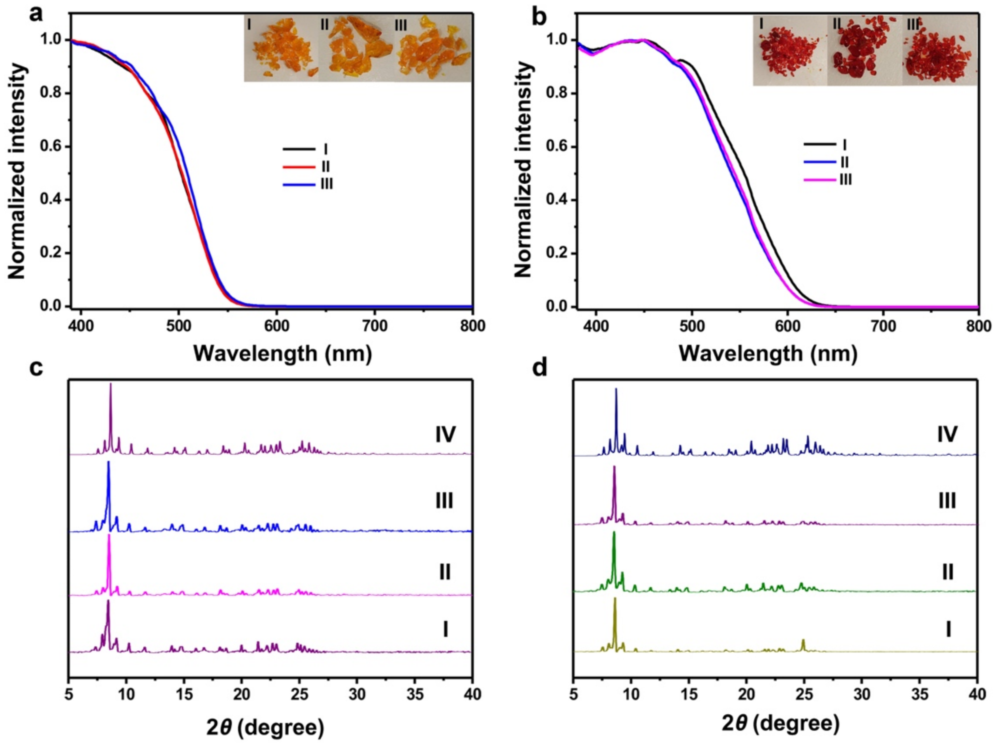

Supplementary Figure 66. (a) Normalized solid-state UV−vis absorption spectra: (I) EtP5-NBNβ, after exposure to (II) 1-BBU vapor and (III) 1-BPE vapor. (b) Normalized solid-state UV−vis absorption spectra: (I) EtP5-DNBγ, after exposure to (II) 1-BBU vapor and (III) 1-BPE vapor. (c) PXRD patterns of EtP5-NBNβ: (I) original EtP5-NBNβ; (II) after exposure to 1-BBU vapor; (III) after exposure to 1-BPE vapor; (IV) simulated from the crystal structure of EtP5-NBNβ. (d) PXRD patterns of EtP5-DNBγ: (I) original EtP5-DNBγ; (II) after exposure to 1-BBU vapor; (III) after exposure to 1-BPE vapor; (IV) simulated from the crystal structure of EtP5-DNBγ.

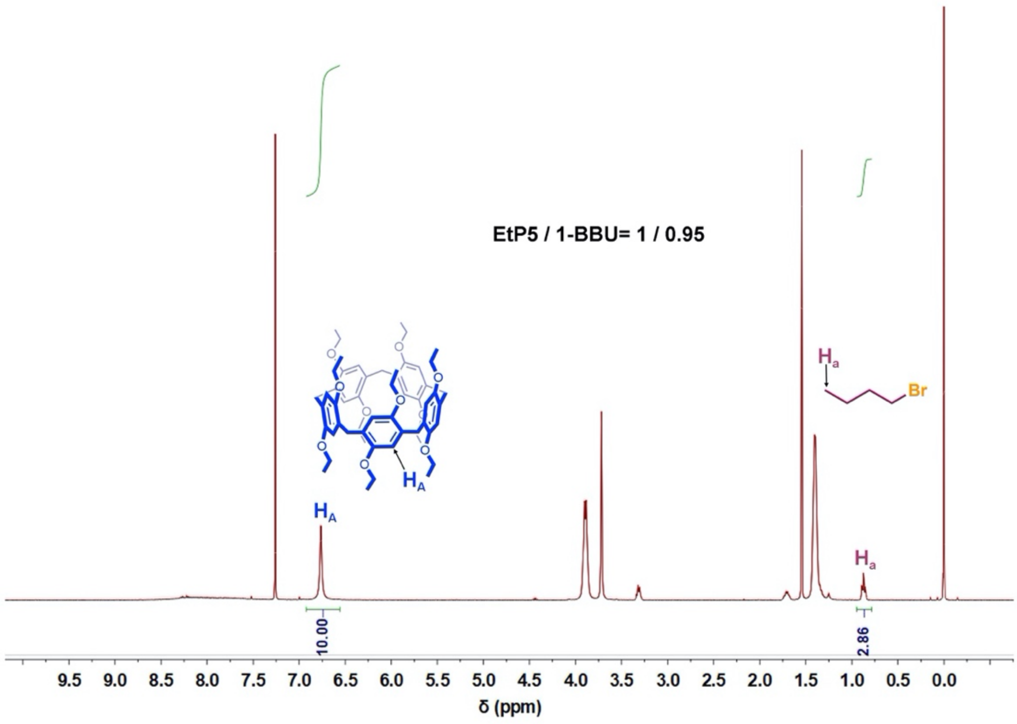

Supplementary Figure 67. ^{1}H NMR spectra (400 MHz, $CDCl_3$, 298 K) of EtP5-NBNα after exposure to 1-BBU/2-BBU mixture (v/v = 1:1) vapor.

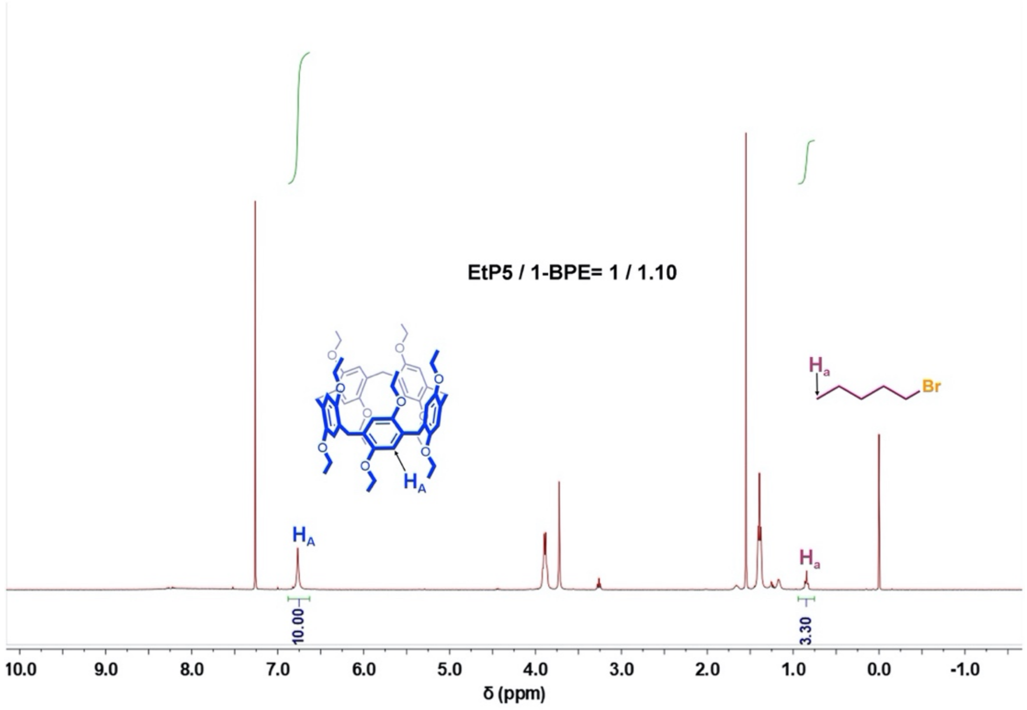

Supplementary Figure 68. ^{1}H NMR spectra (400 MHz, $CDCl_3$, 298 K) of EtP5-NBNα after exposure to 1-BPE/2-BPE mixture (v/v = 1:1) vapor.

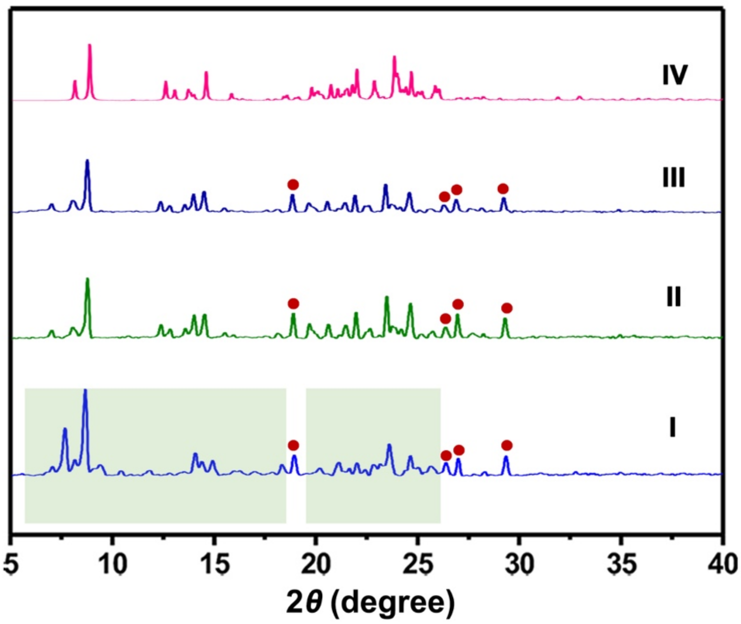

Supplementary Figure 69. PXRD patterns of EtP5-NBNα: (I) original EtP5-NBNα; (II) after exposure to 1-BBU vapor; (III) after exposure to 1-BBU/2-BBU mixture (v/v = 1:1) vapor; (IV) simulated from the crystal structure of 1-BBU⊂EtP5. The red dots represent the peaks of unbonded NBN crystalloids, and the green shades cover the peaks of the D-A complex.

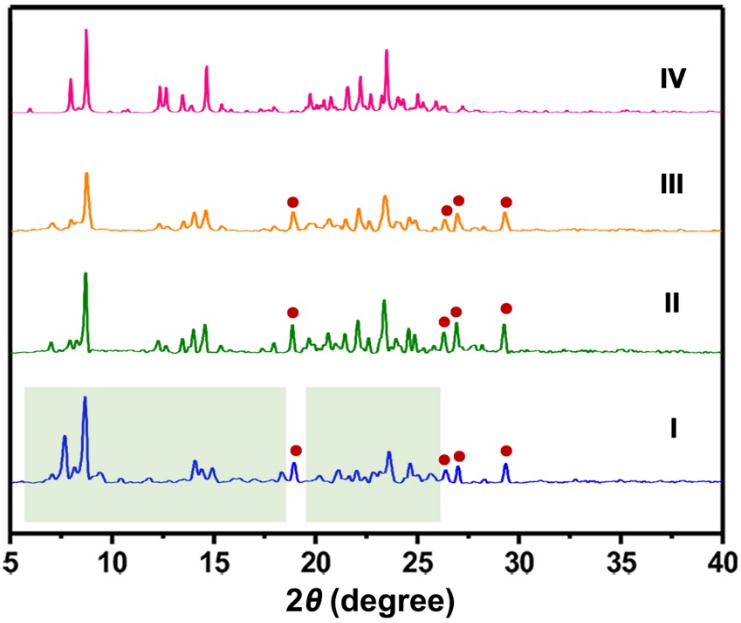

Supplementary Figure 70. PXRD patterns of EtP5-NBNα: (I) original EtP5-NBNα; (II) after exposure to 1-BPE vapor; (III) after exposure to 1-BPE/2-BPE mixture (v/v = 1:1) vapor; (IV) simulated from the crystal structure of 1-BPE⊂EtP5. The red dots represent the peaks of unbonded NBN crystalloids, and the green shades cover the peaks of the D-A complex.

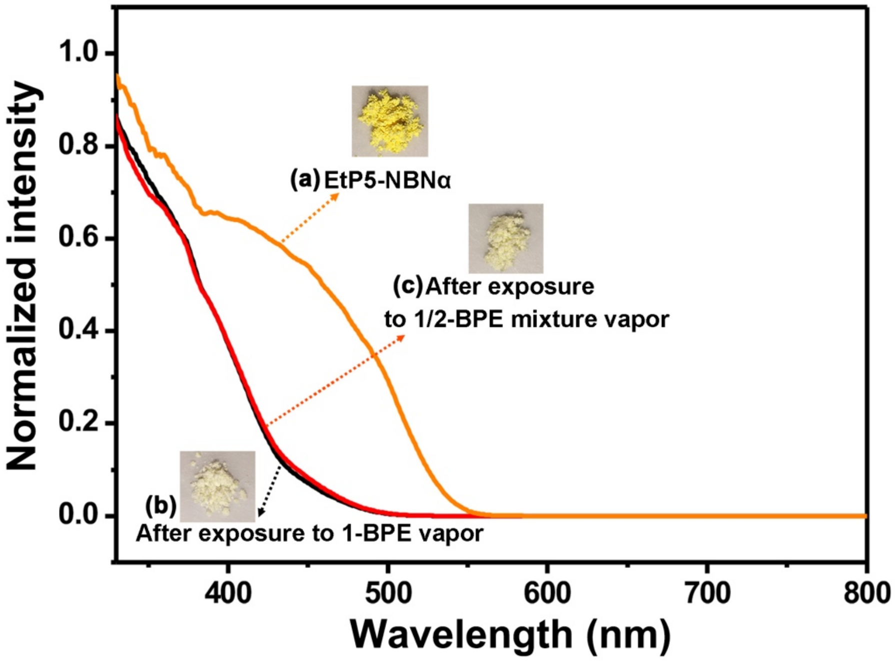

Supplementary Figure 71. Normalized solid-state UV/Vis absorption spectra: (a) original EtP5-NBNα; (b) after exposure to 1-BPE vapor; (c) after exposure to 1-BPE/2-BPE mixture (v/v = 1:1) vapor.

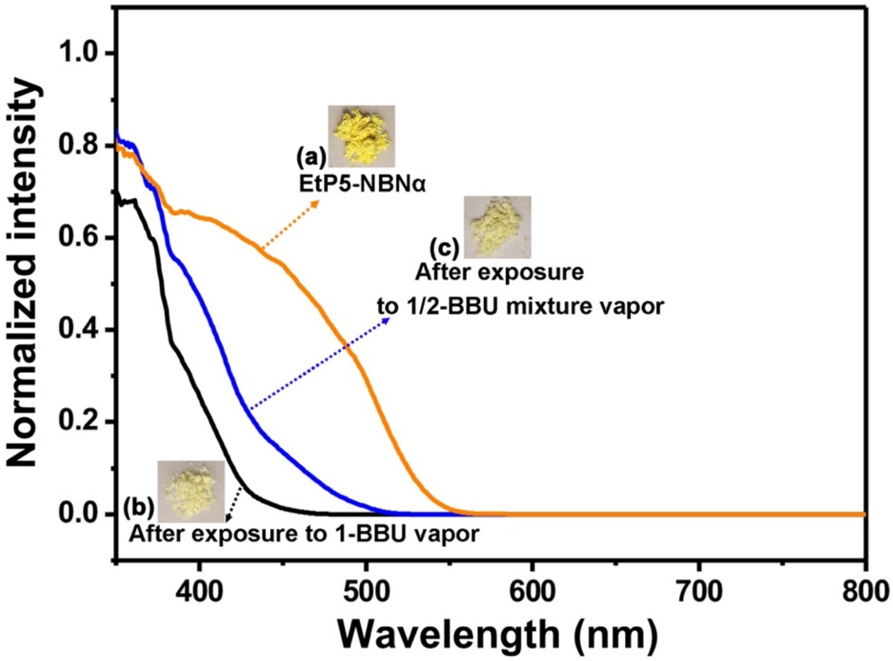

Supplementary Figure 72. Normalized solid-state UV/Vis absorption spectra: (a) original EtP5-NBNα; (b) after exposure to 1-BBU vapor; (c) after exposure to 1-BBU/2-BBU mixture (v/v = 1:1) vapor.

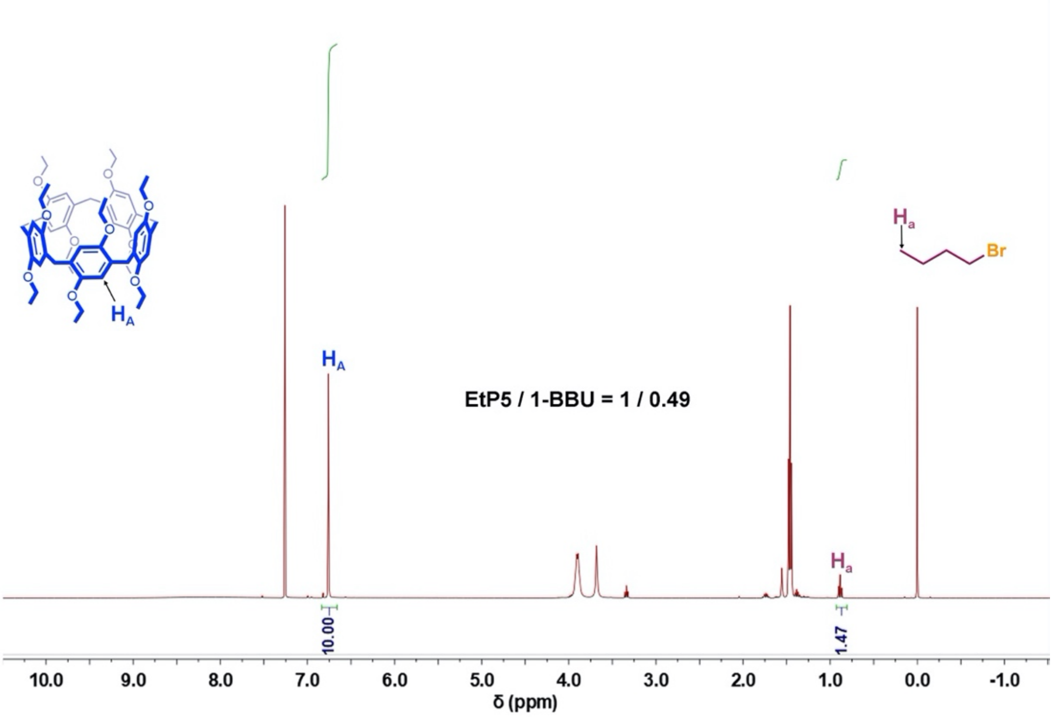

Supplementary Figure 73. ^{1}H NMR spectra (400 MHz, $CDCl_3$, 298 K) of EtP5-DNBα after exposure to 1-BBU/2-BBU mixture (v/v = 1:1) vapor.

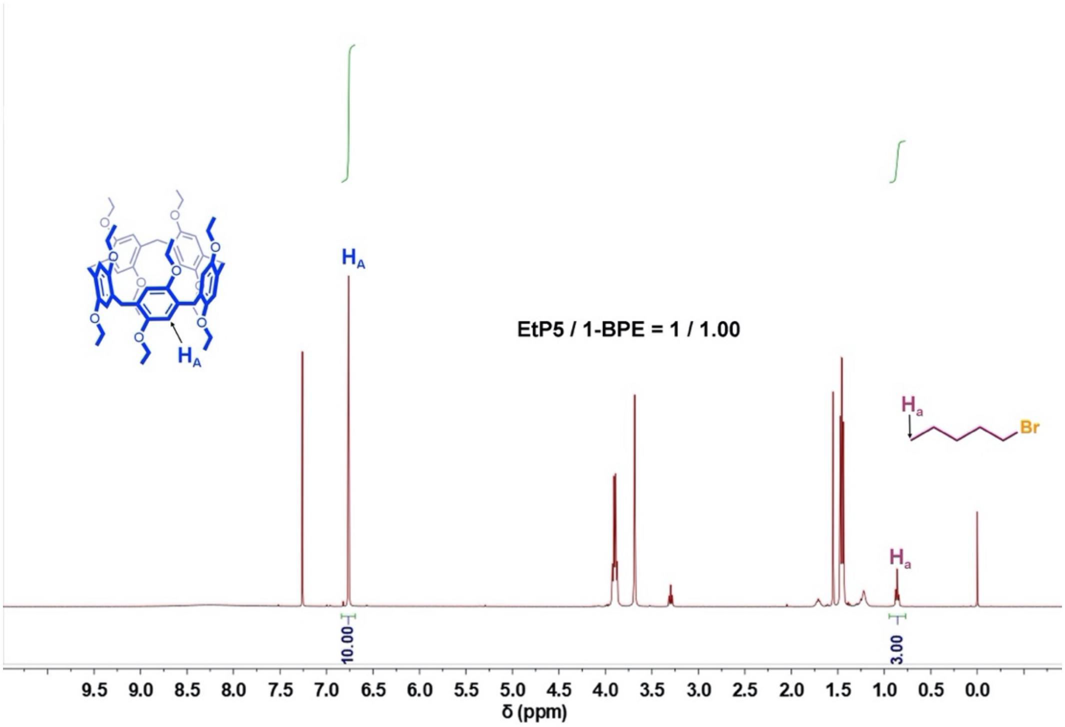

Supplementary Figure 74. ^{1}H NMR spectra (400 MHz, $CDCl_3$, 298 K) of EtP5-DNBα after exposure to 1-BPE/2-BPE mixture (v/v = 1:1) vapor.

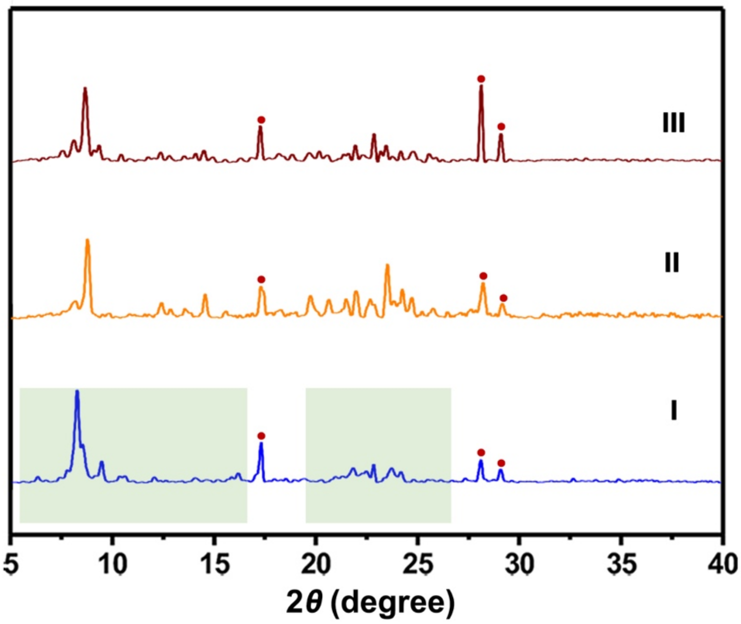

Supplementary Figure 75. PXRD patterns of EtP5-DNBα: (I) original EtP5-DBNα; (II) after exposure to 1-BBU vapor; (III) after exposure to 1-BBU/2-BBU mixture (v/v = 1:1) vapor. The red dots represent the peaks of unbonded DNB crystalloids, and the green shades cover the peaks of the D-A complex.

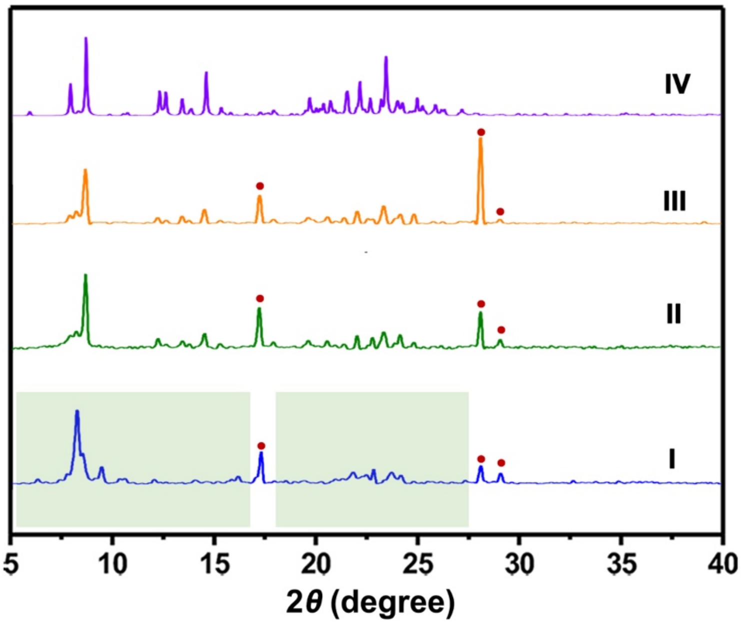

Supplementary Figure 76. PXRD patterns of EtP5-DNBα: (I) original EtP5-DNBα; (II) after exposure to 1-BPE vapor; (III) after exposure to 1-BPE/2-BPE mixture (v/v = 1:1) vapor; (IV) simulated from the crystal structure of 1-BPE⊂EtP5. The red dots represent the peaks of unbonded DNB crystalloids, and the green shades cover the peaks of the D-A complex.

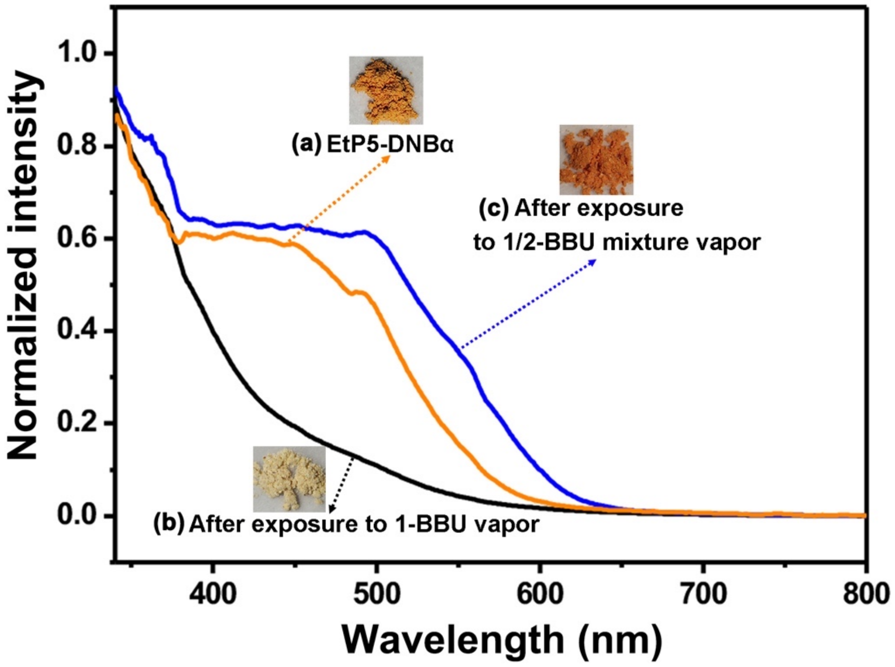

Supplementary Figure 77. Normalized solid-state UV/Vis absorption spectra: (a) original EtP5-DNBα; (b) after exposure to 1-BBU vapor; (c) after exposure to 1-BBU/2-BBU mixture (v/v = 1:1) vapor.

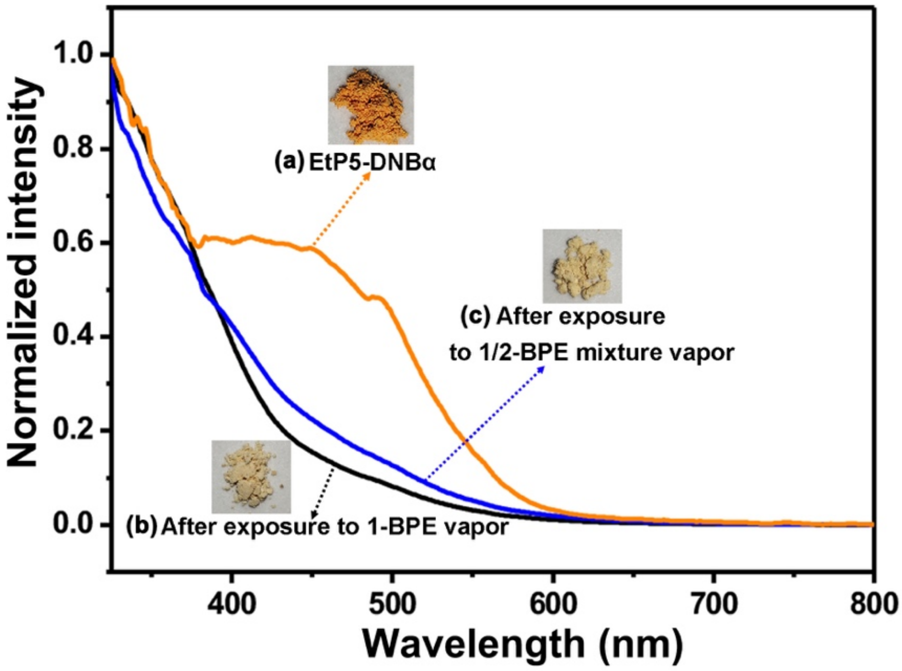

Supplementary Figure 78. Normalized solid-state UV/Vis absorption spectra: (a) original EtP5-DNBα; (b) after exposure to 1-BPE vapor; (c) after exposure to 1-BPE/2-BPE mixture (v/v = 1:1) vapor.

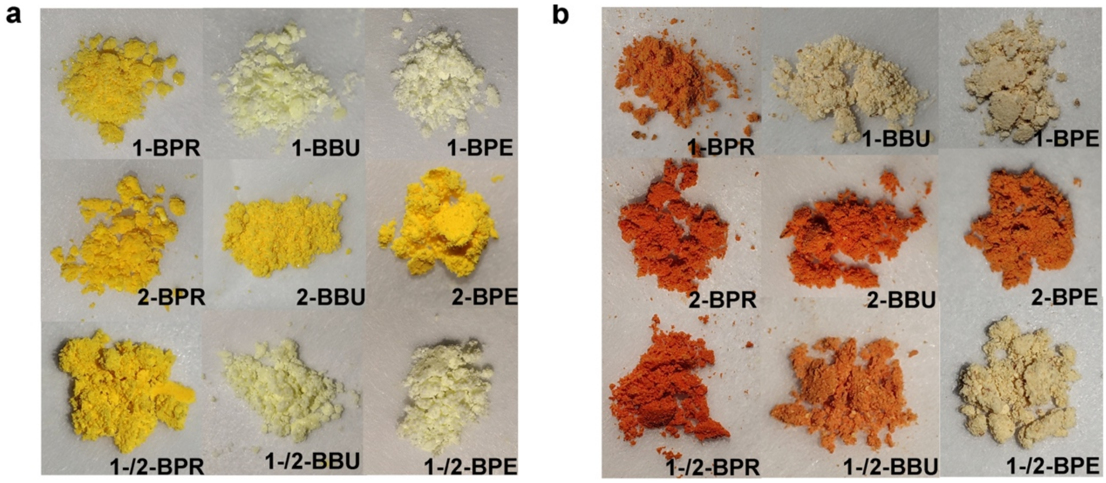

Supplementary Figure 79. Optical images showing the color changes of (a) EtP5-NBNα and (b) EtP5-DNBα upon exposure to different bromoalkane and bromoalkane mixtures.

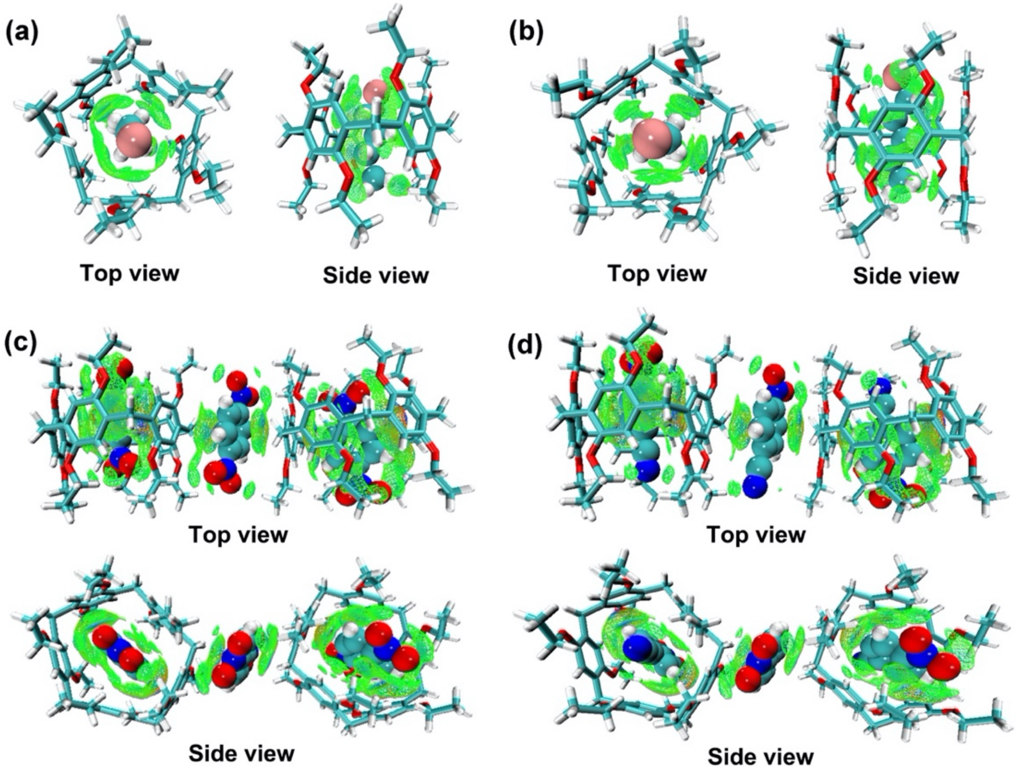

Supplementary Figure 80. Comparison of host–guest binding iso-surfaces (δginter = 0.005) between (a) 1-BBU⊂EtP5 and (b) 1-BPE⊂EtP5, and between (c) EtP5-NBNβ and (d) EtP5-DNBγ.

2.5. Recyclability of EtP5-NBNα and EtP5-DNBα

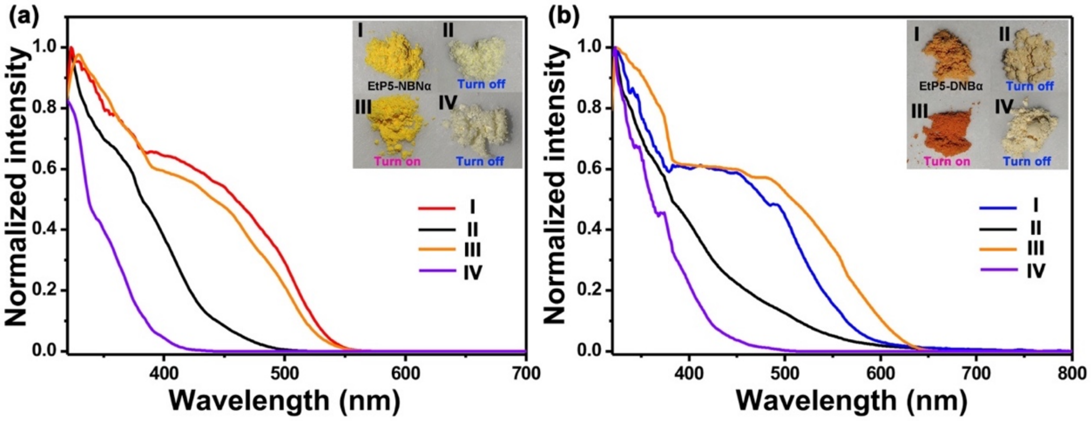

Supplementary Figure 81. (a) Normalized solid-state UV/Vis absorption spectra of EtP5-NBNα: (I) original EtP5-NBNα; (II) after exposure to 1-BPE/2-BPE mixture (v/v = 1:1) vapor; (III) the re-activated CT complex by heating (90 °C) under reduced pressure; (IV) after being exposed to the same mixture vapor again. (b) Normalized solid-state UV/Vis absorption spectra of EtP5-DNBα: (I) original EtP5-DNBα; (II) after exposure to 1-BPE/2-BPE mixture (v/v = 1:1) vapor; (III) the re-activated CT complex by heating (90 °C) under reduced pressure; (IV) after being exposed to the same mixture vapor again. These imply that the CT interactions between EtP5 and the acceptors (NBN and DNB) will be restored upon removal of the trapped 1-BPE in the materials.

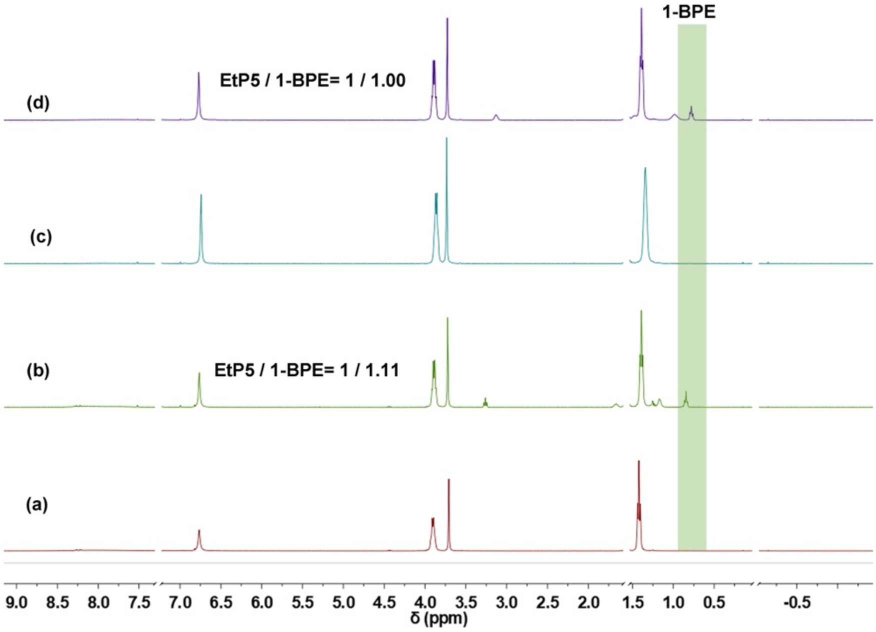

Supplementary Figure 82. ^{1}H NMR spectra (400 MHz, $CDCl_3$, 298 K) of EtP5-NBNα: (a) original EtP5-NBNα; (b) after exposure to 1-BPE/2-BPE mixture (v/v = 1:1) vapor; (c) the re-activated CT complex by heating (90 °C) under reduced pressure; (d) after being exposed to the same mixture vapor again.

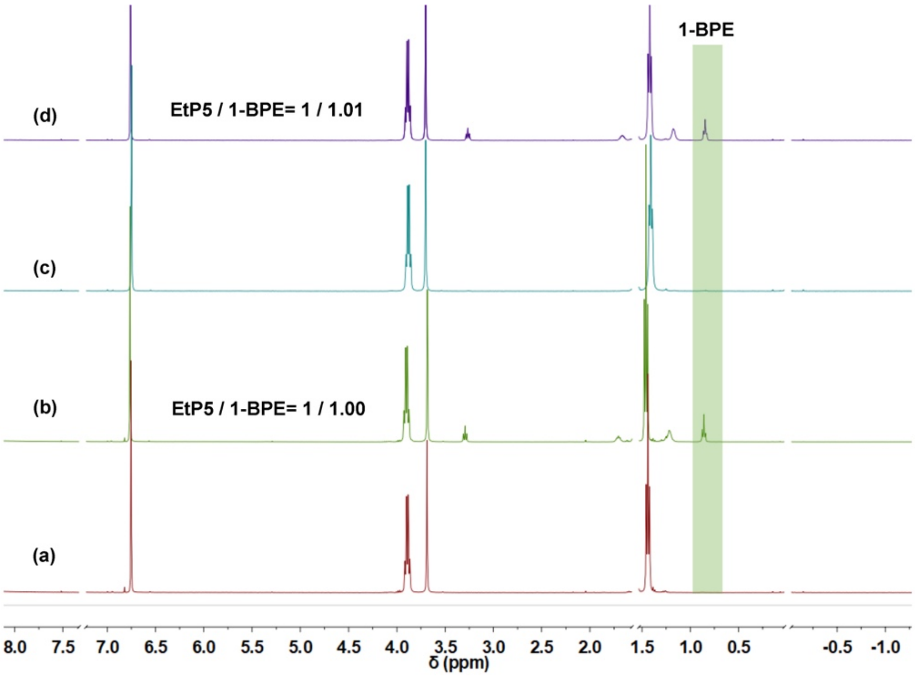

Supplementary Figure 83. ^{1}H NMR spectra (400 MHz, $CDCl_3$, 298 K) of EtP5-DNBα: (a) original EtP5-DNBα; (b) after exposure to 1-BPE/2-BPE mixture (v/v = 1:1) vapor; (c) the re-activated CT complex by heating (90 °C) under reduced pressure; (d) after being exposed to the same mixture vapor again.

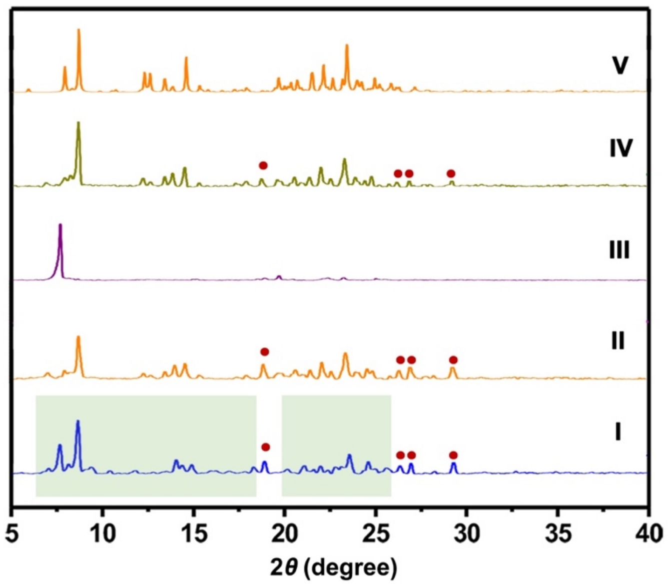

Supplementary Figure 84. PXRD patterns of EtP5-NBNα: (I) original EtP5-NBNα; (II) after exposure to 1-BPE/2-BPE mixture (v/v = 1:1) vapor; (III) the re-activated CT complex by heating (90 °C) under reduced pressure; (IV) after being exposed to the same mixture vapor again; (V) simulated from the crystal structure of 1-BPE⊂EtP5. The red dots represent the peaks of unbonded NBN crystalloids, and the green shades cover the peaks of the D-A complex.

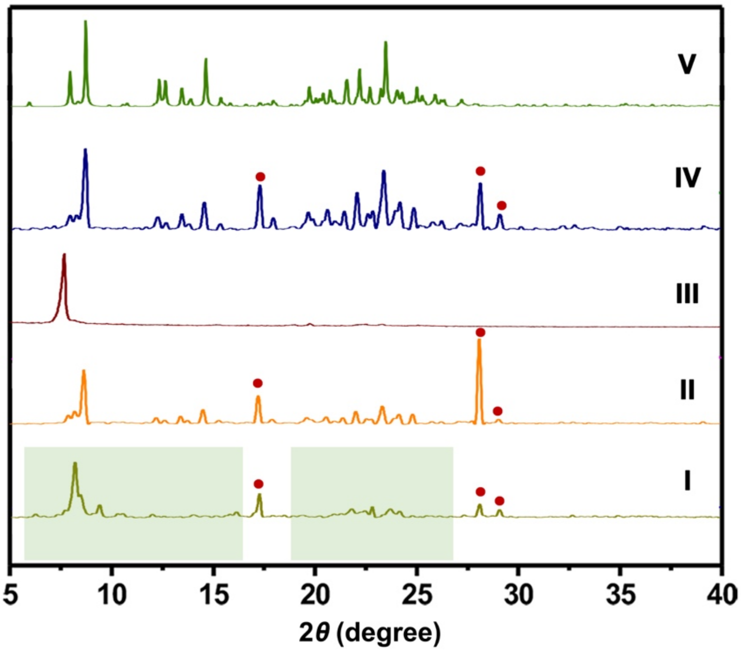

Supplementary Figure 85. PXRD patterns of EtP5-DNBα: (I) original EtP5-DNBα; (II) after exposure to 1-BPE/2-BPE mixture (v/v = 1:1) vapor; (III) the re-activated CT complex by heating (90 °C) under reduced pressure; (IV) after being exposed to the same mixture vapor again; (V) simulated from the crystal structure of 1-BPE⊂EtP5. The red dots represent the peaks of unbonded DNB crystalloids. The red dots represent the peaks of unbonded DNB crystalloids, and the green shades cover the peaks of the D-A complex.

3. Supplementary References

1. Hu X-B, Chen Z, Chen L, Zhang L, Hou J-L, Li Z-T. Pillar[n]arenes (n = 8–10) with two cavities: synthesis, structures and complexing properties. *Chem. Commun.* **48**, 10999-11001 (2012).

2. Sheldrick GM. SHELXT - Integrated space-group and crystal-structure determination. *Acta Crystallogr. A Found. Adv.* **71**, 3-8 (2015).

3. Sheldrick GM. Crystal structure refinement with SHELXL. *Acta Crystallogr. C Struct. Chem.* **71**, 3-8 (2015).

4. Dolomanov OV, Bourhis LJ, Gildea RJ, Howard JAK, Puschmann H. OLEX2: a complete structure solution, refinement and analysis program. *J. Appl. Cryst.* **42**, 339-341 (2009).

5. Lefebvre C, Rubez G, Khartabil H, Boisson JC, Contreras-Garcia J, Henon E. Accurately extracting the signature of intermolecular interactions present in the NCI plot of the reduced density gradient versus electron density. *Phys. Chem. Chem. Phys.* **19**, 17928-17936 (2017).

6. Lu T, Chen FW. Multiwfn: A multifunctional wavefunction analyzer. *J. Comput. Chem.* **33**, 580-592 (2012).

7. Humphrey W, Dalke A, Schulten K. VMD: Visual molecular dynamics. *J. Mol. Graph.* **14**, 33-38 (1996).

8. Thordarson P. Determining association constants from titration experiments in supramolecular chemistry. *Chem. Soc. Rev.* **40**, 1305-1323 (2011).

9. Hibbert DB, Thordarson P. The death of the Job plot, transparency, open science and online tools, uncertainty estimation methods and other developments in supramolecular chemistry data analysis. *Chem. Commun.* **52**, 12792-12805 (2016).

10. Shu X*, et al.* Complexation of neutral 1,4-dihalobutanes with simple pillar[5]arenes that is dominated by dispersion forces. *Org. Biomol. Chem.* **10**, 3393-3397 (2012).
